# Heterogeneity of Vascular Endothelial Cells, De Novo Arteriogenesis and Therapeutic Implications in Pancreatic Neuroendocrine Tumors

**DOI:** 10.3390/jcm8111980

**Published:** 2019-11-14

**Authors:** Bin Ren, J. Bart Rose, Yehe Liu, Renata Jaskular-Sztul, Carlo Contreras, Adam Beck, Herbert Chen

**Affiliations:** 1 Department of Surgery, University of Alabama at Birmingham, Birmingham, AL 35294, USA; jbrose@uabmc.edu (J.B.R.); rjsztul@uabmc.edu (R.J.-S.); ccontreras@uabmc.edu (C.C.); awbeck@uabmc.edu (A.B.); hchen@uabmc.edu (H.C.); 2 O’Neal Comprehensive Cancer Center, University of Alabama at Birmingham, Birmingham, AL 35294, USA; 3 Nutrition & Obesity Research Center, University of Alabama at Birmingham, Birmingham, AL 35294, USA; 4 Diabetes Research Center, University of Alabama at Birmingham, Birmingham, AL 35294, USA; 5 Department of Biomedical Engineering, University of Alabama at Birmingham, Birmingham, AL 35294, USA; 6 Graduate Biomedical Science Program of the Graduate School, University of Alabama at Birmingham, Birmingham, AL 35294, USA; 7 Department of Biomedical Engineering, Case Western Reserve University, Cleveland, OH 44106, USA; yx1448@case.edu

**Keywords:** angiogenesis, arteriogenesis, CD36, cancer stem cells, FoxO1, Notch, protein kinase D, neuroendocrine tumor, transdifferentiation

## Abstract

Arteriogenesis supplies oxygen and nutrients in the tumor microenvironment (TME), which may play an important role in tumor growth and metastasis. Pancreatic neuroendocrine tumors (pNETs) are the second most common pancreatic malignancy and are frequently metastatic on presentation. Nearly a third of pNETs secrete bioactive substances causing debilitating symptoms. Current treatment options for metastatic pNETs are limited. Importantly, these tumors are highly vascularized and heterogeneous neoplasms, in which the heterogeneity of vascular endothelial cells (ECs) and de novo arteriogenesis may be critical for their progression. Current anti-angiogenetic targeted treatments have not shown substantial clinical benefits, and they are poorly tolerated. This review article describes EC heterogeneity and heterogeneous tumor-associated ECs (TAECs) in the TME and emphasizes the concept of de novo arteriogenesis in the TME. The authors also emphasize the challenges of current antiangiogenic therapy in pNETs and discuss the potential of tumor arteriogenesis as a novel therapeutic target. Finally, the authors prospect the clinical potential of targeting the FoxO1-CD36-Notch pathway that is associated with both pNET progression and arteriogenesis and provide insights into the clinical implications of targeting plasticity of cancer stem cells (CSCs) and vascular niche, particularly the arteriolar niche within the TME in pNETs, which will also provide insights into other types of cancer, including breast cancer, lung cancer, and malignant melanoma.

## 1. Introduction

Pancreatic neuroendocrine tumors (pNETs) represent a group of rare neoplasms that originate from pancreatic endocrine cells [1]. They are highly vascularized and heterogeneous neoplasms, which are characterized by high levels of vascular endothelial growth factor (VEGF) and its receptors [2], the potential driver in the metastatic process of pNETs [3], particularly in hepatic metastases [4]. PNETs also highly express platelet-derived growth factor receptors (PDGF-Rs), suggesting an increase in vascular maturation and arteriolar formation in the tumor microenvironment (TME). Sunitinib, a receptor tyrosine kinase (RTK) inhibitor and angiogenesis inhibitor targeting VEGFRs and PDGF-Rs, has been approved for the treatment of advanced pNETs. However, the therapeutic efficacy is limited, accompanied by high rates of progression. Therefore, tailoring antiangiogenic therapy to patients with pNETs requires novel insight into tumor angiogenesis. Recent advances in the understanding of the microenvironment biology of pNETs made VEGF and PDGF pathways interesting targets due to their associations with tumor angiogenesis [5,6], particularly their associations with the development of more mature tumor vessels and arterioles or “de novo arteriogenesis” in the TME. 

Angiogenesis is considered one of the hallmarks in tumor growth and metastasis [7], in which the heterogeneity of vascular endothelial cells (ECs) and de novo arteriogenesis may play important roles and serve as new therapeutic targets, especially in highly angiogenic tumors such as pNETs. John Hunter, a British surgeon, was the first to coin the term angiogenesis by describing blood vessels that grow in reindeer antlers in 1787 [8]. Two centuries later, Dr. Judah Folkman, a surgeon at Harvard Medical School, further developed the concept of angiogenesis, which was defined as the development of new blood vessels from preexisting vessels via sprouting [9]. Mechanistically, angiogenesis is the growth and remodeling process of primitive networks into a complex network [10]. 

Broadly speaking, the growth of new blood vessels includes vasculogenesis, angiogenesis, arteriogenesis and venogenesis. Vasculogenesis is defined as the generation of blood vessels from hemangioblasts (endothelial precursors) during embryonic development of the cardiovascular system [11], including the initial formation of blood islands and tubes. This is followed by the development of vascular trees with the myriad of blood vessels to nourish all tissues and organs. Vasculogenesis can also occur during tumor progression, which may lead to the formation of immature and poorly functioning vascular networks [10]. 

Angiogenesis is a more generic concept referring to the formation of new microvessels [12]. This process is also known as neoangiogenesis under both ischemic and neoplastic conditions [13], where new capillaries are formed by sprouting or longitudinally splitting of preexisting blood vessels [14,15]. The capillary networks are fed by the arterioles, the terminal components of the arterial system via arteriogenesis.

Arteriogenesis refers to a process in which smooth muscle cells (SMCs) cover ECs during vascular myogenesis, accompanied by vascular stabilization. A typical change seen in arteriogenesis is the enlargement of preexisting arterioles [10]. However, an adult arteriogenesis can be a de novo process that occurs by blood vessel expansion and capillary arterialization [16,17,18]. Previous studies suggested that de novo arteriogenesis in adult organisms under ischemic and oncogenic conditions [19,20,21] could be associated with CD36 expression. CD36 is a key regulator in angiogenesis and fatty acid metabolism [22,23] and is a potential driver in metastatic cancer stem cells (CSCs) [24,25,26]. 

Venogenesis is used to define the formation of new venous vessels [27]. Similar to the ECs in the arteriogenesis, the venous ECs may generate different batches or concentrations of similar factors to complete the recruitment and differentiation of venous SMCs and the formation of new venules during angiogenic processes. The venule is the first ramification of the venous system that can drain blood and components in the microcirculation away from the capillary networks. 

**Figure 1:**
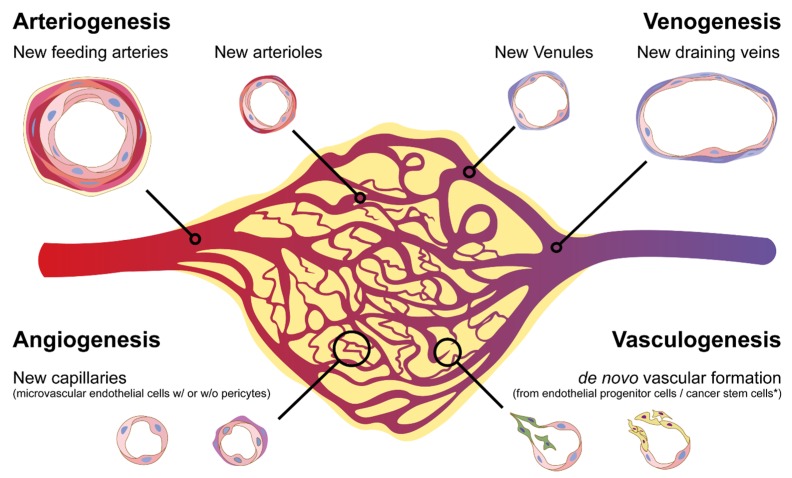
Heterogeneity of blood vessels in the tumor microenvironment. During tumor progression, new blood vascular networks will be developed to provide nutrients and oxygen and remove the metabolic wastes. They can also interact with other types of cells within the vascular niche. Shown is the formation of several types of blood vessels through processes, including de novo arteriogenesis, venogenesis, vascular remodeling, angiogenesis, and vasculogenesis.

As for the tumor vasculature, it is highly heterogeneous with regard to their organization, function, and structure. Six distinct types of tumor-associated blood vessels have been identified in several types of human cancers and replicated in an animal model. These vessels develop into neoangiogenesis by three distinct but parallel interrelated processes: angiogenesis, arteriogenesis, and venogenesis [20,27,28], as well as vasculogenesis by the formation of capillaries via endothelial progenitor cells or cancer stem cells [29,30] (Figure 1). 

These different angiogenic processes may be determined by heterogeneous groups of vascular ECs that constitute the linings of the entire vascular system within TMEs. Therefore, ECs that are heterogeneous in different microenvironments may determine the complexity and diversity of their functions by serving as versatile and multifunctional organs [31,32,33].

## 2. Heterogeneity of Vascular Endothelial Cells

A variety of vascular ECs are the key cell type that constitutes different kinds of blood vessels, and capillary angiogenesis is primarily mediated by the assembly of ECs. However, ECs are extremely heterogeneous. This EC heterogeneity is responsible for the formation of heterogeneous blood vessels and is involved in normal tissue homeostasis and various pathologies. The local microenvironment likely causes phenotypic differences in vascular ECs via genetic, epigenetic, signaling, and cellular differentiation mechanisms [21,32,34]. The morphological, functional, and behavioral heterogeneity existing in the vascular ECs is accompanied by various differential gene expression profiles [19,21,34,35,36,37]. Additionally, ECs can sense and respond to various signals such as changes in local oxygenation, altered hemodynamic forces, and concentrations of signaling molecules from their microenvironment to maintain their functional heterogeneity. Paracrine signaling in the TME can regulate EC gene transcription, allowing for adaptive changes in a tissue-dependent manner. Therefore, the vascular endothelium should be considered as a consortium of distinct individual organs located within blood vessels, which are uniquely adapted to meet the demands of the specific microenvironments [32], such as TMEs. 

EC heterogeneity actually occurs during angiogenic sprouting, where tip cells lead the way, followed by morphologically and functionally distinct stalk cells. During this process, Notch/VEGFR-signaling regulates the differential dynamics of VE-cadherin junctions and drives functional EC rearrangements [38]. Recent studies suggest that ECs such as microvascular ECs (MVECs) may undergo transdifferentiation and change into arteriolar ECs in response to specific environmental cues via stimulation of the protein kinase D (PKD-1) signaling pathway and epigenetic regulation of the activities of transcriptional factor FoxO1 [21,39]. Furthermore, forced expression of PKD-1, an important kinase in VEGF-mediated angiogenesis [40], can induce a tip cell phenotype [35]. These data suggested that certain ECs may inherently have genetic traits of tip cells, which can be induced epigenetically under specific temporal and spatial conditions, thereby coordinating with stalk cells to promote functional angiogenesis [41].

Phenotypic heterogeneity may also occur in tumor endothelium, including that of pNETs. Tumor-associated EC (TAEC) heterogeneity can be additive to the TME, tumor cell, and CSC heterogeneity. The ECs lining the tumor vessels are structurally and functionally abnormal, showing growth properties of cancer cells and projecting into the vessel lumen [42]. The tumor endothelium is covered by morphologically abnormal pericytes and demonstrates increased fenestrations with widened intercellular junctions or gaps. Therefore, the permeability is generally increased in certain tumor-associated vessels [43]. The TME where TAECs are exposed is unique, consisting of cancer and stromal cells such as tumor-associated fibroblasts (TAF) [44] and tumor-associated macrophages (TAM) [45]. Cancer cells may hijack the TAECs, TAFs, and TAMs for their own advantage via paracrine stromal interactions [46]. Moreover, hypoxia significantly stimulates tumor vessel growth by upregulating multiple pro-angiogenic signaling pathways, which can regulate vascular patterning, maturation, and function [47,48]. 

Intriguingly, the stromal cells within the TME may release cytokines, including soluble growth stimulators and inhibitors such as VEGF, FGF-2, thrombospondin-1 (TSP-1), and endostatin. These factors may regulate the behavior of TAECs via interaction with their receptors and influence the angiogenic status during tumor progression [20,39,40,49,50,51,52,53]. The tumor-associated vessels often appear to be more dilated and tortuous, show excessive branching morphogenesis, form arteriovenous shunts, and lack normal artery–capillary–vein hierarchy [54]. However, the vascular networks should have feeding arteriolar vessels, which can provide blood and nutrients in tumor tissues [55] and may also occur in pNETs. Therefore, TAECs may be critical in determining the angiogenic status and formation of the type of blood vessels within the TME. Unlike what researchers originally thought, TAECs are actually not genetically stable but unstable and show a different gene expression profile and respond differently to growth factors compared to normal ECs [56]. This may create complexity but also opportunities to discover new targeted therapy against tumor angiogenesis. 

Added to the complexity of TAEC heterogeneity are the possibilities of CSC transformation into ECs. It has been shown that a sub-population of stem-like cells can generate ECs [30,57] due to the plasticity and transdifferentiation of cancer cells. These abnormal TAECs, in turn, release a variety of factors and cytokines to affect the tumor growth [58,59,60] and possibly to promote tumor metastasis. Moreover, tumors hijack physiological or developmental vascular endothelial processes, including angiogenic sprouting or vasculogenesis. They can develop vascular networks via vessel co-option or intussusception (splitting of pre-existing mother vessels to give rise to daughter vessels), and parasitize the host’s vascular system to promote malignant progression [27,61,62], which may be involved in different TAECs and likely cooperates with stromal vascular cells, TAFs, TAMs, and other immune cells. Thus, TAEC heterogeneity may have a different impact on cancer behavior. Due to the heterogeneity of TAECs, it is a huge challenge to pinpoint a single overall function that defines the EC population. The arteriolar TAECs may express high levels of delta-like 4 (DLL4), a Notch ligand, which can interact with Notch receptors in CSCs to promote CSC maintenance and self-renewal. Therefore, arterial differentiation and arteriolar TAECs could be critical for the development of de novo arteriogenesis under ischemic conditions within the TME of well-vascularized tumors such as pNETs, thereby facilitating metastasis and leading to therapeutic resistance. 

## 3. De Novo Arteriogenesis, an Emerging Concept of Formation of New Vascular Networks 

Angiogenesis, as a hallmark of cancer, supplies oxygen and nutrients and disposes wastes, which is critical for tumor growth and metastatic spreading [9,63,64]. Tumor angiogenesis originally referred to new capillary growth by regeneration of a population of capillary ECs within a neoplasm [63]. Tumor cells cannot grow more than 2–3 mm in diameter without angiogenesis [65]. Tumor angiogenesis is regulated by VEGF prominently via VEGF receptor 2 (VEGFR-2) signaling in vascular ECs [53]. This signaling pathway is also required for angiogenic remodeling [66], an important process of vascular maturation and arteriogenesis. The anti-VEGF monoclonal antibody bevacizumab has shown certain clinical significance in multiple tumor types with limited efficacy, which probably results from its targeting mainly at the newly formed capillaries but not at matured tumor-associated vessels and newly formed tumor-associated arterioles [27] that we call de novo arteriogenesis. 

There is a general belief that arteriogenesis refers to the remodeling process of pre-existing arteries or the increase in the lumen volume and size of the vessel wall, in which smooth muscle cell (SMC) proliferation may play an essential role [10,67,68]. However, de novo arteriogenesis represents the formation of new arteriolar networks via capillary arterialization, in which the proliferation and arteriolar differentiation of ECs, particularly MVECs, may be critical [17,18,19,21,69,70,71,72]. 

EphrinB2 represents the earliest specific marker for arterial ECs [73]. In Zebrafish, the gridlock gene, an HLH gene required for assembly of the aorta, specifies arterial fate [74,75] and regulates the Notch signaling pathway [76,77]. Inhibition of the Notch pathway in ECs by gridlock determines an arterial fate, while VEGF can upregulate the expression of ephrinB2 and stimulates the arterial fate of ECs [78,79,80]. Angiopoietins, a multifaceted cytokine that functions in angiogenesis, also regulates an arterial fate of ECs via modifying VEGF functions [80]. The small chemical molecule GS4898 can rescue the gridlock function in a Zebrafish model with a gridlock mutant phenotype [81,82]. This small chemical molecule promotes arterial differentiation via stimulating the MAPK/Erk pathway during postnatal angiogenesis in a mouse hindlimb ischemia model [19]. These studies suggest a role of de novo arteriogenesis during development and under ischemic conditions. The micro-CT imaging actually documented the occurrence of the newly formed arterioles under ischemic conditions [19].

Recent studies have shown that lysophosphatidic acid (LPA), a lipid signaling mediator, may facilitate the formation of functional arterioles in cooperation with VEGF in vivo [21]. This biological effect may be associated with FoxO-1 regulation of VEGF expression and crosstalk between VEGF signaling and the CD36 pathway [39]. Studies suggest that MVECs may be converted to arteriolar ECs. This process is likely to be involved in the CD36-mediated priming of VEGF signaling and capillary arterialization [22,35,40,41,83]. In fact, the crosstalk between angiogenic and antiangiogenic signaling could be critical to the specification of arterial ECs [39,40].

Venous ECs can be converted to arterial ECs by VEGF both in vitro and in vivo [79,80], further exemplifying the plasticity of vascular EC phenotypes. This phenomenon is supported by the fact that shear stress in circulation may determine the phenotypes of ECs [84], leading to the formation of either arterioles or venules through differentiation of two distinct types of ECs. 

Vascular ECs are indeed critical for the regulation of arteriogenesis. In response to VEGF and other cytokines, ECs can be activated to increase the expression of FGF-2, platelet-derived growth factor PDGF-B and TGF-β1, thereby inducing the regrowth of SMCs and vessel enlargement [10,85]. Moreover, VEGF-mediated arteriogenic gene expression and Notch signaling may be essential for arterial differentiation and arteriolar remodeling in the TME [19,21,35,66], and may determine the arterial fate and stimulate de novo arteriogenesis via preferential activation of downstream MAPK/Erk rather than PI3Kinase/Akt signaling as shown in animal models [19,81]. We propose that during adult angiogenesis, arteriolar ECs can signal recruitment and appropriate differentiation of arteriolar SMCs, thus leading to the development of arterioles, particularly under ischemic and oncological conditions. Furthermore, arteriolar ECs will generate a variety of factors, including PDGF-B, TGF-β1, FGF-2, and thrombospondin 1 (TSP-1) to facilitate the recruitment and proliferation of arteriolar SMCs to form arterioles. This is accompanied by a corresponding formation of the extracellular matrix, leading to the development of a mature arteriolar network. 

The arterioles that feed into a capillary network in the TME [55] represent a long-term structural adaptation to the altered metabolic demand [86], likely occurring via de novo arteriolar remodeling of capillaries into arterioles [17,19,21,70,87,88]. The significant increase in intratumoral capillaries during tumor progression [89,90] reasonably requires the concurrent expansion of upstream arterioles [20,55,89,91]. The analysis of tumor angiogenesis based on TAEC proliferation and pericyte recruitment demonstrated that there is active angiogenesis in several types of human tumors [92]. The results actually implicate the formation of feeding arterioles or de novo arteriogenesis [20,27,28] since the staining for the tumor vessels was not confirmed with other specific markers other than α-SMA, a key marker for SMCs [92]. Dr. Harold Dvorak’s group elegantly documented the appearance of arteries and arterioles in the TME [27]. 

Most tumors continue to generate a significant amount of VEGF over long periods of time, thus, continually inducing the formation of new blood vessels [27]. In collaboration with LPA and/or FGF-2, the VEGF might concurrently lead to previously formed vessels to develop into more stable forms of arteriolar vasculature [21,85,93] within the TME. In response to VEGF overexpression, capillaries are enlarged and transformed toward an arterial phenotype in a process that is known as capillary arterialization [94] or arteriogenesis. Similarly, Dvorak’s group showed that in the TME of VEGF-secreting tumors, Ad-VEGF-A164 stimulates abnormal arteriogenesis and venogenesis via remodeling of pre-existing arteries and veins to feed and drain the angiogenic vascular bed in animal models [27].

In fact, extensive studies show that arteriogenesis may likely occur within the TME in animal models and in patients with cancer [21,27,28,55,88,89,91,95,96,97], possibly within the TME of pNETs. NETs, including pNETs, classically, are most easily apparent in the early arterial phase of a computed tomography (CT) scan. For decades, it has been clinically appreciated that many primary gastrointestinal NETs and metastatic sites have a pattern of early arterial enhancement on cross-sectional imaging. Consequently, contrasted multiphase CT or magnetic resonance imaging is an important component in the evaluation of a patient with suspected primary or recurrent NETs [98,99]. Compared with normal pancreatic islets, pNETs have increased expression of nestin, probably contributing to vascular remodeling within the TME of pNETs [27,28,55,100]. Though the vessels in grade 3, NETs display the highest EC angiogenic activity, and they have regained pericyte coverage [101]. These studies suggest an increase in the formation of matured blood vessels and possibly the development into arterioles within the TME of pNETs. The development of arteriogenesis is supported by studies showing the high levels of pro-arteriogenic factors VEGF, VEGF receptors, and FGF-2 in NETs, but not in normal islet cells. Moreover, recent studies suggest that MVEC transdifferentiation into arteriolar ECs is likely an approach for facilitating the formation of arterioles under physiological or pathological conditions [20,21]. Intriguingly, during development, different types of blood vessels may be generated from different origins. Pulmonary capillaries are developed by angiogenesis, while pulmonary arteries are developed by vasculogenesis [102], which further supports the concept that de novo arteriogenesis exists under physiological and pathological conditions [19,20]. 

Maturation of the endothelial networks within the TME involves remodeling and ‘pruning’ capillary-like vessels with uniform size, and irregular organization into a structured network of branching vessels. Blood flow in tumor vessels is often chaotic, slow, and not efficient in meeting metabolic demands in some tumors [103]. However, blood vessels in tumor beds should be functional enough to allow oxygen and nutrients to be supplied and metabolic wastes to be removed. De novo arteriogenesis may be the case in highly angiogenic pNETs, in which the antiangiogenic drug sunitinib is partially effective as a targeted therapy against tumor vessels [104]. 

## 4. Tumor Arteriogenesis: Potential Target in pNETs

Tumor angiogenesis has been extensively studied since Folkman coined this concept more than three decades ago [63], whereas the role of de novo arteriogenesis within the TME is important but under-appreciated, and the mechanisms remain largely unknown. The arterioles to supply the vascular beds of tumors [55] might be generated by de novo arteriogenesis. The arteriolar differentiation of TAECs (a key component of the CSC niche [105]) and arteriolar remodeling within the TME might serve as a unique vascular niche for CSC maintenance and self-renewal in malignant progression of pNETs and other types of cancers, including breast and lung cancers, and malignant melanoma. 

Actually, not only do ECs serve as gatekeepers of organ homeostasis [106], but they are also essential to maintain the function of arterioles in providing nutrients to cancer cells [19,20,107], including CSCs. EC differentiation likely plays a key role in tumor arteriogenesis [20,27,55,91,108] in that arteriolar ECs may recruit SMCs to form arterioles and promote tumor progression by serving as an arteriolar niche for CSC maintenance and self-renewal. Prior studies have shown that the LPA/PKD-1-CD36 signaling axis switches MVECs to an “arteriolar phenotype” [21,71,109]. We postulated that TAECs also possess plasticity and may be reprogrammed for arteriolar differentiation toward arteriolar remodeling in response to microenvironmental factors within the TME for the progression of pNETs. 

NETs are regarded as neoplasms originating in the hormone-producing cells of the endocrine system. PNETs represent 1–2% of all pancreatic tumors and 7% of NETs in general, second only to gastrointestinal carcinoid [110,111,112]. Current studies suggest that NETs may derive from mature neuroendocrine cells that undergo dedifferentiation due to genetic mutations or the progenitors of the neuroendocrine cells that undergo mutations, and even from the non-neuroendocrine cells that acquire neuroendocrine characteristics during carcinogenesis due to the loss of certain genes [113]. These studies indicate that the cellular plasticity contributes to the formation of NETs, including pNETs, and suggests the existence of CSC plasticity.

PNETs can be hormonal non-functioning or functioning. Up to 30% of tumors can secrete bioactive substances, including insulin, gastrin, glucagon, and somatostatin [114]. PNETs are often asymptomatic and grow slowly over several years before becoming symptomatic from mass effect, with a favorable five-year relative survival rate of 54% across all Surveillance, Epidemiology, and End Results (SEER) stages combined (www.cancer.org/cancer/pancreatic-neuroendocrine-tumor). However, a small, poorly differentiated subset is associated with a very aggressive phenotype [115]. More than 10% of pNETs present hepatic metastases upon diagnosis based on several multi-center studies [116], and approximately 85% of patients will develop hepatic metastases during a follow-up period of 20 years [117]. Current surgical and medical management have limited efficacy in the metastatic setting and show a poor prognosis compared to resected local-only disease [118,119]. Intriguingly, pNETs are highly vascularized and heterogeneous neoplasms [120], which are characterized by high levels of VEGF and its receptors [2]. Cytokine VEGF is a potential driver in the metastatic process of pNETs [3], particularly in hepatic metastases [4]. PNETs also highly express PDGFRs, which promote vascular maturation and arteriogenesis [121]. Both VEGFRs and PDGFRs are targets for the RTK sunitinib, which has shown limited antiangiogenic effects though the drug can increase progression-free survival by six months only in some patients with advanced pNETs [122,123]. 

Antiangiogenic therapy may show clinical benefits in patients with pNETs with a high intensity of neoangiogenesis [124]. The antiangiogenic drug bevacizumab was tested in phase II studies of advanced pNETs in combination with octreotide and chemotherapy with acceptable toxicity but showed limited improvement [125,126]. The limited response in these early studies may be a result of limited VEGF targeting that only inhibits the growth of the newly formed capillaries in the TME but has limited action against stable tumor vasculature [127]. 

Studies implicate that arteriolar formation or de novo arteriogenesis may play a critical role in tumor progression [20,55]. The concept that targeting arterioles improves prognosis is supported by the fact that the inhibition of the PDGF pathway enhances the efficacy of agents targeting VEGF [128] since PDGFs promote vascular maturation and arteriogenesis by mediating the recruitment of pericytes or SMCs to the newly formed blood vessels [10]. LPA likely promotes de novo arteriogenesis in the TME [20]. It is reasonable to speculate that in the early phases of cancer progression, the neoangiogenesis is dependent more on the VEGF pathway than PDGF and LPA signaling. However, this VEGF dependency may be reduced or lost in later phases when mature blood vessels are present [27]. It is during this later phase that arteriogenic drivers such as PDGF and LPA are likely more important because of their leading to angiogenic inhibitor-resistance [20,21,28,128,129]. Therefore, it is essential to establish appropriate animal cancer models that can mimic human cancer vasculature, including the formation of later stage and matured vascular networks, feeding arterioles, and drainage veins. Better understanding pathways that regulate tumor arteriogenesis may be a fundamental step to discovering an approach for controlling the progression of different types of cancer, including pNETs. 

## 5. Antiangiogenic Therapy in pNETs: Challenges and Prospective

PNETs often present with metastases. Conventional therapies for advanced disease are rarely curative, and cytoreductive surgery showed limited results [130]. Targeting angiogenesis in pNETs results in therapeutic resistance. However, targeted antiangiogenic therapies like sunitinib are promising treatment options in the clinic. A phase III trial comparing sunitinib to placebo in well differentiated, unresectable pNETs showed a significant benefit in progression free survival (PFS) and suggested an improvement in overall survival [122]. Five-year follow-up of this study confirmed a benefit in PFS but failed to show an improvement in overall survival, possibly confounded by cross-over [104]. However, a phase IV trial supports the outcome of the phase III trial, further confirming that sunitinib is an efficacious and safe treatment option [131]. 

A recent study analyzed a small cohort of pNET patients with sunitinib treatment in a real-world clinical setting [132]. In this clinical scenario, over 98.8% had received three or more therapeutic regimens before initiating sunitinib therapy. Among those patients, 80% had been treated with everolimus, and 95% with somatostatin analogs. Additionally, it appears that there was a synergic effect between somatostatin analogs and sunitinib based on median PFS. This study demonstrated that sunitinib was safe and effective in the clinical setting even if the patients were undergone pre-treatment. However, the significance is limited due to a retrospective investigation of a low number of patients.

Everolimus (an mTOR inhibitor) as a target agent is also effective in advanced, well-differentiated progression pNETs [133]. The RADIANT-3 trial found that everolimus also significantly prolonged progression-free survival among patients with low or intermediate grade pNETs with progressive advanced disease [133]. However, everolimus may only show a limited effect in targeting tumor angiogenesis when compared with sunitinib. Furthermore, both sunitinib and everolimus are non-specific and may target many different types of cells and show severe side-effects. 

Somatostatin analogs have shown antitumor effects via direct inhibition of cellular proliferation and tumor progression and indirect regulation of angiogenesis in gastroenteropancreatic neuroendocrine tumors [134]. In a randomized, double-blind study, lanreotide, the somatostatin analog, was used in patients with metastatic enteropancreatic NETs and showed prolonged progression-free survival through mechanisms that were not completely understood [135]. Early in 1991, somatostatin analogs were reported to inhibit angiogenesis of normal vascular development in vitro [136]. Their antiangiogenic activity in the TME was elegantly discussed in the literature about tumor angiogenesis [137,138], possibly via acting on the VEGF pathway in TAECs [139,140]. 

Current antiangiogenic therapy is based on the concept that the molecular phenotype of neoangiogenesis in the TME is active and immature, in which VEGF is a key player. Researchers try to target these immature vessels selectively, without affecting the quiescent organ vasculature [141,142,143,144]. Unfortunately, tumors often evade current single-agent antiangiogenic therapy via the induction of alternative proangiogenic pathways [3,145]. Moreover, some tumors even increase invasiveness and metastasis in response to current antiangiogenic therapy [48,146]. 

VEGF is considered as a key driver in the metastatic process of pNETs [3]. Targeting the VEGF pathway shows antitumor effects in mouse models of pNETs. However, human patients will potentially become resistant over time and develop metastases [147], which may be associated with FGF-associated intratumor hypoxia [3,146]. Moreover, hypoxia-induced hypoxia-inducible factor (HIF)-1α or HIF-2α stimulates the formation of normalized functioning vessels [48]. This also supports the existence of arteriogenesis within the TME [28,90,91] and suggests that the formation of functional arterioles within the TME in pNETs may play an important role in malignant progression and liver metastasis. Intriguingly, concomitant inhibition of c-Met and VEGF signaling has synergistic effects in pNETs [6], indicating that inhibition of multiple signaling pathways may increase the efficacy of anti-angiogenesis drugs. Recently, clinical trials are undergoing to investigate the combinations of immunotherapy with anti-angiogenesis treatment [148]. However, to maximize the efficacy of anti-angiogenesis, one widely adopted strategy is to combine drugs targeting multiple pathways in angiogenesis. We will focus on the Notch signaling pathway and vascular niche and CSC plasticity. 

### 5.1. Notch Pathway: The Potential Target in pNETs

Expression of Notch receptors and ligands continue to be present in mature vessels to maintain vascular integrity and homeostasis [149], whereas inhibition of DLL4/Notch signaling enhances non-functional vessel growth and significantly limits tumor growth by reducing blood perfusion in lung malignancies [150]. DLL4-mediated Notch signaling is also critical during active vascularization, but less important for the maintenance of normal blood vessels [151]. Blocking DLL4 signaling inhibits tumor growth, which may be associated with defective maturation of the tumor vascular network and poor tissue perfusion [152]. These studies, thus, support the concept that functional angiogenesis and arteriolar networks in the TME may promote malignant progression. 

Intriguingly, forced expression of DLL4 in tumor cells affects the morphogenesis of tumor vasculature, leading to the non-functional angiogenesis in the TME [150]. DLL4 expression in myotubes can activate Notch3 in adjacent myoblasts, thereby signaling those cells for quiescence [153]. This suggests that the DLL4/Notch axis restores the SC pool via SC self-renewal. Because arteriolar ECs express DLL4 and CSCs express Notch receptors, it is reasonable to hypothesize that this crosstalk exists between TAECs in the arterioles and CSCs within the TME. Moreover, this interaction may be critical for CSC maintenance and self-renewal in pNETs. Blocking DLL4 signaling may disrupt the crosstalk of arteriolar ECs with CSCs via targeting the Notch pathway. Therefore, the combination of sunitinib with anti-DLL4 can be a potential strategy to increase therapeutic efficacy. However, to develop an optimal therapeutic strategy, it is imperative to have a better understanding of the mechanisms of tumor arteriogenesis and the oncogenic signaling essential for pNET progression.

Currently, little is known about the degree of active angiogenesis and the functional status of the vasculature within human cancers, including pNETs. The optimal vascular network should be highly organized, including venules, capillaries, and arterioles, to supply all cells with sufficient nutrients. This may not be the case in the TME for different types of cancers, but a similar organization could exist in pNETs as the early arterial phase of a CT scan can easily show pNETs and targeting the arteriogenic pathway by sunitinib had certain therapeutic effects. 

Additionally, the prognostic value of microvascular density (MVD) in NETs is controversial: some studies showed that low MVD and high endothelial proliferation index are unfavorable prognostic values [154], whereas other studies suggest that MVD was by no means a prognostic factor in pNETs [155]. In low-grade pNETs, the blood vessels are well branched, and the total surface area is expanded, whereas vessels in high-grade pNETs are plump, non-branched, and accompanied by a decreased vessel surface [156]. Increased blood vessel size or altered shape may be a marker of poor prognosis in other cancers such as squamous cell carcinoma of the vulva [157]. The observation, thus, suggests that the functional status and formation of arterioles or de novo arteriogenesis could be an important prognostic index and contribute to the progression of pNETs. 

Moreover, the Notch pathway may be a key regulator of both tumor angiogenesis [150] and tumorigenesis, specifically in NETs [158,159]. Prior studies [160,161,162,163,164] have shown that Notch1 signaling is minimal or absent in pulmonary, thyroid, adrenal, and pNETs. Overexpression of Notch1 in a human pNET cell-line (BON cells) using an inducible construct demonstrated the suppression of tumor growth, an increase in the downstream target of the Notch1 Hes, a decrease in NET marker ASCL1 [161], and inhibition of neuroendocrine differentiation [164]. The inhibition of differentiation suggests that Notch1 signaling may promote the development of the stemness of CSCs in pNETs, which is supported by another study [165]. In contrast, a tissue microarray of 120 well-differentiated NETs arising from the pancreas (*n* = 74) reveals significant variability in Notch1 signaling across different tissue types, with an elevated Notch1 expression in 34% of human pNETs [166]. In patients with well-differentiated pNETs, 43.7% demonstrated positive Notch1 expression [167], suggesting heterogeneous expression of this pathway in pNETs. Moreover, Notch1 signaling shows significant variability in tumor status across different tissue types, which may promote or inhibit tumor progression [168,169]. Further studies are needed to elucidate the mechanisms of Notch1 in pNET progression.

Intriguingly, another Notch isoform, Notch3, has been shown to inhibit the progression of medullary thyroid carcinoma [170,171] and may be a therapeutic target [172]. Notch3 inhibits the progression of small cell lung cancer [173], likely as a result of deregulated Notch functions in cell fate decisions [174]. Functionally, Notch may serve as either an oncogene or a tumor suppressor, depending on cellular context. Targeting Notch/Notch-ligands interplay may be an effective strategy for pNETs. DLL3 inhibits Notch activation by interfering with DLL1/Notch interaction, leading to failure of cell membrane translocation. A DLL3 monoclonal antibody conjugated with a toxic chemotherapeutic agent appears to be an effective therapeutic strategy in preclinical models of small cell lung cancer [175]. The novel DLL3-targeting antibody-drug conjugate Rovalpituzumab is currently being utilized in clinical trials [176]. Antibody-drug conjugates targeting DLL3 are able to eliminate CSCs in xenograft models of pulmonary neuroendocrine cancers [177]. These studies indicate that significant genetic and epigenetic heterogeneity could exist among different types of NETs in terms of expression of Notch receptors and target genes. It is worthy of noting that the Notch pathway is known as the key regulator of tumor angiogenesis and arterial differentiation, a key process in de novo arteriogenesis [19,21,39,71,178]. Therefore, elucidating the mechanisms by which Notch signaling crosstalk between arteriolar ECs and CSCs of pNETs in the vascular niche may hold the key to successful therapeutic approaches to targeting this pathway in both cancer and vascular compartments at the same time.

### 5.2. Control of pNET Progression by Targeting Vascular Niche and CSC Plasticity

Emerging evidence supports that tumor recurrence and metastasis are driven by a subpopulation of CSCs that can undergo self-renewal and differentiation. Intact tumors harbor CSCs in dedicated niches (microenvironment) [179,180,181,182,183,184]. Intriguingly, the plasticity of this cell population allows them to alternate between CSC and non-CSCs, representing a substantial difference when compared to normal SCs. [185]. These phenotypic transitions can be driven by environmental stimuli [186,187,188,189,190]. A new and emerging concept indicates that CSCs may be much more plastic and abundant and can proliferate vigorously [191,192,193], rather than presenting as hierarchies as normal SCs. This plasticity in CSCs may be an important driver of drug resistance via change of its status into quiescence. Interestingly, pancreatic adenocarcinoma xenografts appear not to be driven by quiescent CSCs but rather by the successive activation of transiently active CSCs [193]. Both differentiated and transient amplifying cancer cells can be reprogrammed into CSCs if appropriate niche signals exist in the TME [185]. On the other hand, genetic mutations may occur to sustain the self-renewal of CSCs during tumor progression and render CSCs progressive independent of niche signals. An autonomous CSC phenotype can significantly increase numbers of CSCs within tumor tissue via blocking differentiation [185]. While CSCs have also been shown to be present in pNETs [165], it is not clear what degree of CSCs plasticity exists in this disease. Given the intratumor plasticity on top of the inherent mutability of cancer cells [194], it may be an attractive therapeutic strategy to modulate stem cell-niche functions such as vascular niche rather than to pursue therapies that are only based on intrinsic CSC features in order to keep CSC dormant or induce its differentiation into normal cells. 

Moreover, mitochondrial respiration may play an important role in the maintenance and self-renewal of CSCs [195]. Like quiescent muscle SCs, which are dependent on oxidative phosphorylation mainly through mitochondrial fatty acid oxidation [196], quiescent CSCs might also rely on oxidative metabolism via fatty acid oxidation for their maintenance. Inhibition of oxidative phosphorylation and fatty acid metabolism could deplete this cell population, thereby improving responses to chemotherapeutics and targeted therapies. This was shown in mouse models of pancreatic cancer [197,198,199]. To achieve optimal therapeutic responses, targeting CD36, a fatty acid receptor and antiangiogenic receptor, could be beneficial because CD36 not only inhibits tumor angiogenesis and is associated with arteriolar differentiation and remodeling [20,21,108,200] but is also expressed in a subpopulation of highly aggressive disseminated CSCs to increase metastatic potential [25]. CD36 as a fatty acid receptor may facilitate the uptake and metabolism of fatty acids in CSCs, possibly representing an opportunity to treat the late stages of disease by controlling CD36-mediated metastasis [25,26,201,202] and by inhibiting arteriogenesis in TMEs. 

Muscle SCs (satellite cells) are deeply quiescent, yet they localize to aerobic niches close to capillary vessels and use oxidative phosphorylation mainly through mitochondrial fatty acid oxidation [196]. Inhibition of fatty acid uptake and metabolism via CD36 indeed inhibits metastasis, though it does not affect the growth of primary oral squamous carcinomas [25]. The arterioles in the TME can provide free oxygen to tumor tissues and could serve as a unique vascular niche for the maintenance and self-renewal of CSCs in a variety of cancers, such as pNETs and breast cancer, that can rely on fatty acid as energy sources. However, the most aggressive forms of CSCs become independent of normal niche signals [190,203,204]. Therefore, it is worthy of studying the arteriolar differentiation and remodeling as a unique arteriolar niche in promoting CSC aggressiveness. 

Furthermore, transcription factors may be involved in CSC stemness and plasticity via the regulation of CD36 transcription. The forkhead box O (FoxO) transcription factors are required for the maintenance of somatic and CSCs [205,206,207], whereas FoxO1 regulates CD36 transcription [21], which could play a key role in the development of metastatic CSCs. Moreover, FoxO1 may be an important player in the formation of a vascular niche by acting on ECs. In blood vessels, FoxO1 is highly expressed in vascular ECs, including MVECs, and is essential for the regulation of EC stability, vascular development, angiogenesis, and arteriolar differentiation [21,200,208,209]. FoxO1 also functions as a potent regulator of adult vascular homeostasis [208] and endothelial quiescence via a coordinated reduction in the proliferative and metabolic activity of vascular ECs [209]. In a Zebrafish model, FoxO1 is able to restore oxidative stress-compromised vascular regeneration via interaction with the Notch pathway [41]. In MVECs and TAECs, FoxO1 can regulate the transcriptional expression of CD36 in response to LPA/PKD-1 signaling [20,21]. These studies strongly suggest the role of FoxO1 in arteriolar differentiation and vascular maturation and implicate the involvement of FoxO1 in the formation of functional arterioles [20,21,39] via priming proangiogenic VEGF signaling [20,21,22,35,39,83]. Therefore, FoxO1 might not only play an important role in the maintenance of the CSC pool but also mediate the formation of a unique vascular niche for the maintenance and self-renewal of CSCs in cancers, including pNETs. 

**Figure 2:**
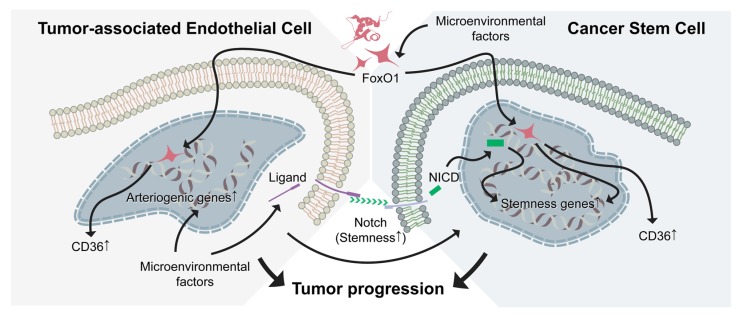
FoxO-1-CD36 and Notch signaling in tumor-associated endothelial cells (TAECs) and cancer stem cells (CSCs). Targeting both the FoxO1-CD36 signaling and Notch pathway may inhibit TAECs and attenuate maintenance and self-renewal of CSCs. Shown in this figure are the upregulated CD36 expression in both TAECs and CSCs via FoxO1. Whereas, the Notch pathway may upregulate arteriogenic genes or Notch ligands in TAECs, thus promoting interactions between TAECs and CSCs. Inhibition of CD36 may attenuate the generation of metastatic CSCs. Combined inhibition of the Notch pathway with CD36 signaling, both arteriogenic TAECs and CSCs will be targeted, subsequently leading to tumor regression and inhibition of tumor metastasis.

Therefore, future potential therapeutic strategies may include combinations of antiangiogenic therapy with anti-CSC strategy by targeting both the FoxO-1-CD36 signaling axis and Notch pathways (Figure 2). This combination might significantly limit the growth of cancers, including pNETs, and inhibit their metastasis by targeting both arteriolar niche and CSCs despite the caveats that CSC plasticity evokes toward the design of anti-CSC therapies. Additionally, venous components could be involved in the regulation of CSC behavior via venogenesis, the functional role of which needs to be further investigated in pNETs but is not discussed in this review. It will also be worthy of a better understanding of the mechanisms by which the vascular niche within the TME specifies the CSC state and plasticity in the setting of pNETs. Moreover, developing clinically relevant pNET models with robust angiogenesis, matured vasculature, and arteriogenesis in animals should facilitate the understanding of mechanisms and early diagnosis. 

The transdifferentiation of TAECs and CSCs of pNETs may be explored and targeted since TAEC heterogeneity may respond to antiangiogenic drugs differently, and the CSC plasticity concept represents the capacity of CSCs undergoing both differentiation and transdifferentiation. Because targeting vascular niche may reactivate and sensitize quiescent CSCs to anti-cancer therapy, an approach to targeting both vascular niche and CSC compartment may present an attractive strategy via the identification of key regulators of arteriolar differentiation and CSC metabolism and differentiation in cancers, including pNETs. In this regard, the PKD-1/CD36-FoxO1 signaling axis is likely to be a promising and potential candidate target. Dissecting this pathway will facilitate the identification of key and targetable regulators because of its close association with both tumor neoangiogenesis (de novo arteriogenesis) and stemness and plasticity of CSCs.

In summary, pNETs exhibit a wide range of biologic behaviors ranging from long dormancy to rapid progression. Currently, only limited therapeutic options are available to patients with advanced pNETs. The efficacy of systemic therapy is poor, with high progression rates. It is well-known that pNETs are highly angiogenic and heterogeneous, with variable prognosis. Sunitinib is the only targeted therapy approved for pNETs, and relapse after an initial response. Carrasco et al., recently reviewed classical antiangiogenic therapies, and discussed some new angiogenic targets in NETs [210]. Here, we summarize some novel antiangiogenic “drug” molecules/agents, which may be promising and potential therapeutic targets in pNETs (Table 1).

Many alternative pathways are involved in the formation of tumor vasculature, which may be independent of VEGF signaling, such as vascular mimicry formation and vascular intussusception. We need to identify the key regulator of heterogeneous tumor vascular networks in the development of pNETs. We also urgently need to elucidate mechanisms by which pNETs progress, relapse, and discover key signaling molecules in de novo arteriogenesis in order to identify new targets for effective therapy. Due to the complexity and dynamic properties of heterogeneous TAECs, we may consider personalized antiangiogenic therapies, likely in combination with checkpoint-inhibitor immunotherapy, based on a patient’s specific profile of tumor tissues and TAECs in order to achieve optimal therapeutic responses in the clinic [230]. Furthermore, the feeding arterioles that are derived from de novo arteriogenesis or the mature, SMC-coated daughter blood vessels could be potentially valuable targets because they supply the smaller, angiogenic vessels enclosed within the tumor mass [19,20,27]. A better mechanistic understanding of angiogenesis and de novo arteriogenesis within the TME, and the role of CSCs in pNET progression may provide insight into novel and effective treatment strategies not only in pNETs but also other highly angiogenic and arteriogenic cancers. 

## Figures and Tables

**Table 1:** Drugs/molecules/agents for potential antiangiogenic treatment in pNETs.

Name	Mechanisms & Functions	Reference(s)
**CVM-1118**	Phase II clinical trial for patients with advanced NETs, including pNETs; de novo development of vascular networks via vascular mimicry, which is associated with a malignant phenotype and a poor clinical outcome. Vascular mimicry was presented in pNET animal models.	https://clinicaltrials.gov/ct2/show/NCT03600233; Chu et al., *Am J Pathol 2013*; Wagenblast, et al., *Nature* 2015; Hendrix, et al., *Pharmacol Ther* 2016 [211,212,213]
**Cabozantinib**	Phase III clinical trial of in patients with advanced neuroendocrine tumors after progression on everolimus (CABINET) A VEGFR, c-MET and AXL inhibitor; attenuate sunitinib therapy-mediated pro-metastasis in xenograft mouse tumor models of renal cell carcinoma.	https://clinicaltrials.gov/ct2/show/NCT03375320; Zhou et al., *Oncogene*, 2015 [214]
**Lenvatinib**	A multi-kinase inhibitor with a preferential antiangiogenic activity.	Capdevila, et al. *J Clin Oncol*, 2019; Capozzi, et al., *Cancer Manag Res* 2019 [215,216]
**TH-302 (evofosfamide)**	A hypoxia-activated prodrug, which is metabolized to its active form, bromo-isophosphoramide mustard (Br-IPM), under hypoxic conditions. Used in combination with sunitinib.	Grande, et al., abstract. *J Clin Oncol*, 2019 [217]
**Doublecortin-like kinase 1 (DCLK1)**	A potential marker for pNET CSCs; induce epithelial-mesenchymal transition (EMT).	Ikezono, et al., *Mol Cancer Res*, 2017 [218]
**MEDI3617**	A monoclonal antibody targeting angiopoietin-2; in combination with VEGF-targeted therapies.	Rigamonti, et al., *Cell Rep* 2014; Biel, et al., *Cancer Letters*, 2016 [219,220]
**Sema3A** **(Semaphorin 3A)**	Overcome cancer hypoxia and metastatic dissemination induced by sunitinib treatment in mice. In combination with sunitinib, Sema3A synergistically enhanced RIP-Tag2 mouse survival.	Maione, F, et al., *J Clin Invest,* 2012 [221]
**Brivanib**	A dual FGF/VEGF inhibitor; vascular inhibition and tumor stability.	Allen, et al., *Clin Cancer Res*, 2011 [222]
**PlGF signaling inhibitor?**	High PIGF expression in pNET patients with poor outcome; overcome resistance to antiangiogenic factors.	Fischer et al., *Cell,* 2007; Hilfenhaus, et al., *Endocr Relat Cancer*, 2013 [223,224]
**EZH2 inhibitor?**	Sensitize tumor to sunitinib in cell lines and PDX models.	Marconcini, et al., 2016 [225]
**Autotaxin (ATX) inhibitors? LPA receptor-specific antagonists?**	High ATX expression in pNET tissues, which was associated with higher tumor grade, TNM staging and lymph node metastasis; ATX drives LPA expression, which is also linked to tumor angiogenesis and arteriolar differentiation and malignancy of tumor cells.	*Liu* et al., *Cancer Cell*, 2009; Chen, et al., *Front Biosci (Landmark Ed)* 2013; Ren, et al., *Arterioscler Thromb Vasc Biol,* 2016; Liu et al., *Cancer Lett* 2016; Dong et al., *Oncotarget*, 2017 [20]; Yang et al., *Endocr. Connect*, 2018 [21,226,227,228,229]

## References

[1] Halfdanarson T.R., Rabe K.G., Rubin J., Petersen G.M. (2008). Pancreatic neuroendocrine tumors (PNETs): Incidence, prognosis and recent trend toward improved survival. Ann. Oncol..

[2] Terris B., Scoazec J.Y., Rubbia L., Bregeaud L., Pepper M.S., Ruszniewski P., Belghiti J., Flejou J., Degott C. (1998). Expression of vascular endothelial growth factor in digestive neuroendocrine tumours. Histopathology.

[3] Casanovas O., Hicklin D.J., Bergers G., Hanahan D. (2005). Drug resistance by evasion of antiangiogenic targeting of VEGF signaling in late-stage pancreatic islet tumors. Cancer Cell.

[4] Gomez D., Malik H.Z., Al-Mukthar A., Menon K.V., Toogood G.J., Lodge J.P., Prasad K.R. (2007). Hepatic resection for metastatic gastrointestinal and pancreatic neuroendocrine tumours: Outcome and prognostic predictors. HPB (Oxford).

[5] De Dosso S., Grande E., Barriuso J., Castellano D., Tabernero J., Capdevila J. (2013). The targeted therapy revolution in neuroendocrine tumors: In search of biomarkers for patient selection and response evaluation. Cancer Metastasis Rev..

[6] Sennino B., Ishiguro-Oonuma T., Wei Y., Naylor R.M., Williamson C.W., Bhagwandin V., Tabruyn S.P., You W.K., Chapman H.A., Christensen J.G. (2012). Suppression of tumor invasion and metastasis by concurrent inhibition of c-Met and VEGF signaling in pancreatic neuroendocrine tumors. Cancer Discov..

[7] Hanahan D., Weinberg R.A. (2011). Hallmarks of cancer: The next generation. Cell.

[8] Lenzi P., Bocci G., Natale G. (2016). John Hunter and the origin of the term “angiogenesis”. Angiogenesis.

[9] Folkman J. (1995). Angiogenesis in cancer, vascular, rheumatoid and other disease. Nat. Med..

[10] Carmeliet P. (2000). Mechanisms of angiogenesis and arteriogenesis. Nat. Med..

[11] Shalaby F., Rossant J., Yamaguchi T.P., Gertsenstein M., Wu X.F., Breitman M.L., Schuh A.C. (1995). Failure of blood-island formation and vasculogenesis in Flk-1-deficient mice. Nature.

[12] Dvorak H.F., Brown L.F., Detmar M., Dvorak A.M. (1995). Vascular permeability factor/vascular endothelial growth factor, microvascular hyperpermeability, and angiogenesis. Am. J. Pathol..

[13] Bernardi R., Guernah I., Jin D., Grisendi S., Alimonti A., Teruya-Feldstein J., Cordon-Cardo C., Simon M.C., Rafii S., Pandolfi P.P. (2006). PML inhibits HIF-1alpha translation and neoangiogenesis through repression of mTOR. Nature.

[14] Potente M., Gerhardt H., Carmeliet P. (2011). Basic and therapeutic aspects of angiogenesis. Cell.

[15] Carmeliet P., Jain R.K. (2011). Molecular mechanisms and clinical applications of angiogenesis. Nature.

[16] Simons M., Ware J.A. (2003). Therapeutic angiogenesis in cardiovascular disease. Nat. Rev. Drug Discov..

[17] Mac Gabhann F., Peirce S.M. (2010). Collateral capillary arterialization following arteriolar ligation in murine skeletal muscle. Microcirculation.

[18] Ren B., Best B., Weihrauch D., Jones D.W., Dong L., Opansky C., Yuan R., Pritchard K.A., Silverstein R. (2016). Abstract 15673: LPA/PKD-1-FoxO1-CD36 Signaling Axis Regulates Capillary Arterialization in Ischemic Conditions. Circulation.

[19] Ren B., Deng Y., Mukhopadhyay A., Lanahan A.A., Zhuang Z.W., Moodie K.L., Mulligan-Kehoe M.J., Byzova T.V., Peterson R.T., Simons M. (2010). ERK1/2-Akt1 crosstalk regulates arteriogenesis in mice and zebrafish. J. Clin. Invest..

[20] Dong L., Yuan Y., Opansky C., Chen Y., Aguilera-Barrantes I., Wu S., Yuan R., Cao Q., Cheng Y.C., Sahoo D. (2017). Diet-induced obesity links to ER positive breast cancer progression via LPA/PKD-1-CD36 signaling-mediated microvascular remodeling. Oncotarget.

[21] Ren B., Best B., Ramakrishnan D.P., Walcott B.P., Storz P., Silverstein R.L. (2016). LPA/PKD-1-FoxO1 Signaling Axis Mediates Endothelial Cell CD36 Transcriptional Repression and Proangiogenic and Proarteriogenic Reprogramming. Arter. Thromb. Vasc. Biol..

[22] Ren B., Hale J., Srikanthan S., Silverstein R.L. (2011). Lysophosphatidic acid suppresses endothelial cell CD36 expression and promotes angiogenesis via a PKD-1-dependent signaling pathway. Blood.

[23] Aitman T.J., Glazier A.M., Wallace C.A., Cooper L.D., Norsworthy P.J., Wahid F.N., Al-Majali K.M., Trembling P.M., Mann C.J., Shoulders C.C. (1999). Identification of Cd36 (Fat) as an insulin-resistance gene causing defective fatty acid and glucose metabolism in hypertensive rats. Nat. Genet..

[24] Yuan Y., Kohlenberg J.D., Chen Y., Komas S., Xin G., Yuan G., Cui W., Wu S., Ren B. (2015). Abstract A09: Diet-induced obesity promotes breast cancer progression by LPA-signaling-mediated functional changes of mitochondria and angiogenesis. Cancer Res..

[25] Pascual G., Avgustinova A., Mejetta S., Martin M., Castellanos A., Attolini C.S., Berenguer A., Prats N., Toll A., Hueto J.A. (2017). Targeting metastasis-initiating cells through the fatty acid receptor CD36. Nature.

[26] Hale J.S., Otvos B., Sinyuk M., Alvarado A.G., Hitomi M., Stoltz K., Wu Q., Flavahan W., Levison B., Johansen M.L. (2014). Cancer stem cell-specific scavenger receptor CD36 drives glioblastoma progression. Stem Cells.

[27] Nagy J.A., Dvorak H.F. (2012). Heterogeneity of the tumor vasculature: The need for new tumor blood vessel type-specific targets. Clin. Exp. Metastasis.

[28] Sitohy B., Nagy J.A., Jaminet S.C., Dvorak H.F. (2011). Tumor-surrogate blood vessel subtypes exhibit differential susceptibility to anti-VEGF therapy. Cancer Res..

[29] Ribatti D. (2004). The involvement of endothelial progenitor cells in tumor angiogenesis. J. Cell Mol. Med..

[30] Wang R., Chadalavada K., Wilshire J., Kowalik U., Hovinga K.E., Geber A., Fligelman B., Leversha M., Brennan C., Tabar V. (2010). Glioblastoma stem-like cells give rise to tumour endothelium. Nature.

[31] Yuan L., Chan G.C., Beeler D., Janes L., Spokes K.C., Dharaneeswaran H., Mojiri A., Adams W.J., Sciuto T., Garcia-Cardena G. (2016). A role of stochastic phenotype switching in generating mosaic endothelial cell heterogeneity. Nat. Commun..

[32] Regan E.R., Aird W.C. (2012). Dynamical systems approach to endothelial heterogeneity. Circ. Res..

[33] Ren B. (2015). Endothelial Cells: A Key Player in Angiogenesis and Lymphangiogenesis. MOJ Cell Sci. Rep..

[34] Chi J.T., Chang H.Y., Haraldsen G., Jahnsen F.L., Troyanskaya O.G., Chang D.S., Wang Z., Rockson S.G., van de Rijn M., Botstein D. (2003). Endothelial cell diversity revealed by global expression profiling. Proc. Natl. Acad. Sci. USA.

[35] Best B., Moran P., Ren B. (2018). VEGF/PKD-1 signaling mediates arteriogenic gene expression and angiogenic responses in reversible human microvascular endothelial cells with extended lifespan. Mol. Cell Biochem..

[36] Nolan D.J., Ginsberg M., Israely E., Palikuqi B., Poulos M.G., James D., Ding B.S., Schachterle W., Liu Y., Rosenwaks Z. (2013). Molecular signatures of tissue-specific microvascular endothelial cell heterogeneity in organ maintenance and regeneration. Dev. Cell.

[37] Marcu R., Choi Y.J., Xue J., Fortin C.L., Wang Y., Nagao R.J., Xu J., MacDonald J.W., Bammler T.K., Murry C.E. (2018). Human Organ-Specific Endothelial Cell Heterogeneity. iScience.

[38] Bentley K., Franco C.A., Philippides A., Blanco R., Dierkes M., Gebala V., Stanchi F., Jones M., Aspalter I.M., Cagna G. (2014). The role of differential VE-cadherin dynamics in cell rearrangement during angiogenesis. Nat. Cell Biol..

[39] Ren B. (2018). FoxO1 transcriptional activities in VEGF expression and beyond: A key regulator in functional angiogenesis?. J. Pathol..

[40] Ren B. (2016). Protein Kinase D1 Signaling in Angiogenic Gene Expression and VEGF-Mediated Angiogenesis. Front. Cell Dev. Biol..

[41] Baek K.I., Packard R.R.S., Hsu J.J., Saffari A., Ma Z., Luu A.P., Pietersen A., Yen H., Ren B., Ding Y. (2018). Ultrafine Particle Exposure Reveals the Importance of FOXO1/Notch Activation Complex for Vascular Regeneration. Antioxid. Redox Signal..

[42] Denekamp J. (1982). Endothelial cell proliferation as a novel approach to targeting tumour therapy. Br. J. Cancer.

[43] Morikawa S., Baluk P., Kaidoh T., Haskell A., Jain R.K., McDonald D.M. (2002). Abnormalities in pericytes on blood vessels and endothelial sprouts in tumors. Am. J. Pathol..

[44] Orimo A., Weinberg R.A. (2006). Stromal fibroblasts in cancer: A novel tumor-promoting cell type. Cell Cycle.

[45] Franklin R.A., Liao W., Sarkar A., Kim M.V., Bivona M.R., Liu K., Pamer E.G., Li M.O. (2014). The cellular and molecular origin of tumor-associated macrophages. Science.

[46] Ronca R., Van Ginderachter J.A., Turtoi A. (2018). Paracrine interactions of cancer-associated fibroblasts, macrophages and endothelial cells: Tumor allies and foes. Curr. Opin. Oncol..

[47] Wilson W.R., Hay M.P. (2011). Targeting hypoxia in cancer therapy. Nat. Rev. Cancer.

[48] Krock B.L., Skuli N., Simon M.C. (2011). Hypoxia-induced angiogenesis: Good and evil. Genes Cancer.

[49] Senger D.R., Galli S.J., Dvorak A.M., Perruzzi C.A., Harvey V.S., Dvorak H.F. (1983). Tumor cells secrete a vascular permeability factor that promotes accumulation of ascites fluid. Science.

[50] Ren B., Hoti N., Rabasseda X., Wang Y.Z., Wu M. (2003). The antiangiogenic and therapeutic implications of endostatin. Methods Find. Exp. Clin. Pharm..

[51] Ren B., Yee K.O., Lawler J., Khosravi-Far R. (2006). Regulation of tumor angiogenesis by thrombospondin-1. Biochim. Biophys. Acta.

[52] Ren B., Song K., Parangi S., Jin T., Ye M., Humphreys R., Duquette M., Zhang X., Benhaga N., Lawler J. (2009). A double hit to kill tumor and endothelial cells by TRAIL and antiangiogenic 3TSR. Cancer Res..

[53] Simons M., Gordon E., Claesson-Welsh L. (2016). Mechanisms and regulation of endothelial VEGF receptor signalling. Nat. Rev. Mol. Cell Biol..

[54] Nagy J.A., Chang S.H., Shih S.C., Dvorak A.M., Dvorak H.F. (2010). Heterogeneity of the tumor vasculature. Semin. Thromb. Hemost..

[55] Dvorak H.F. (2015). Tumor Stroma, Tumor Blood Vessels, and Antiangiogenesis Therapy. Cancer J..

[56] Hida K., Hida Y., Amin D.N., Flint A.F., Panigrahy D., Morton C.C., Klagsbrun M. (2004). Tumor-associated endothelial cells with cytogenetic abnormalities. Cancer Res..

[57] Ricci-Vitiani L., Pallini R., Biffoni M., Todaro M., Invernici G., Cenci T., Maira G., Parati E.A., Stassi G., Larocca L.M. (2010). Tumour vascularization via endothelial differentiation of glioblastoma stem-like cells. Nature.

[58] Samson T., Welch C., Monaghan-Benson E., Hahn K.M., Burridge K. (2010). Endogenous RhoG is rapidly activated after epidermal growth factor stimulation through multiple guanine-nucleotide exchange factors. Mol. Biol. Cell.

[59] Franses J.W., Edelman E.R. (2011). The evolution of endothelial regulatory paradigms in cancer biology and vascular repair. Cancer Res..

[60] Franses J.W., Baker A.B., Chitalia V.C., Edelman E.R. (2011). Stromal endothelial cells directly influence cancer progression. Sci. Transl. Med..

[61] Jain R.K., Carmeliet P. (2012). SnapShot: Tumor angiogenesis. Cell.

[62] Jain R.K. (2014). Antiangiogenesis strategies revisited: From starving tumors to alleviating hypoxia. Cancer Cell.

[63] Folkman J. (1971). Tumor angiogenesis: Therapeutic implications. N. Engl. J. Med..

[64] Pluda J.M. (1997). Tumor-associated angiogenesis: Mechanisms, clinical implications, and therapeutic strategies. Semin. Oncol..

[65] Brem H., Folkman J. (1975). Inhibition of tumor angiogenesis mediated by cartilage. J. Exp. Med..

[66] Hainaud P., Contreres J.O., Villemain A., Liu L.X., Plouet J., Tobelem G., Dupuy E. (2006). The role of the vascular endothelial growth factor-Delta-like 4 ligand/Notch4-ephrin B2 cascade in tumor vessel remodeling and endothelial cell functions. Cancer Res..

[67] Heil M., Eitenmuller I., Schmitz-Rixen T., Schaper W. (2006). Arteriogenesis versus angiogenesis: Similarities and differences. J. Cell Mol. Med..

[68] Cai W.J., Koltai S., Kocsis E., Scholz D., Kostin S., Luo X., Schaper W., Schaper J. (2003). Remodeling of the adventitia during coronary arteriogenesis. Am. J. Physiol.-Heart Circ. Physiol..

[69] Lanahan A., Zhang X., Fantin A., Zhuang Z., Rivera-Molina F., Speichinger K., Prahst C., Zhang J., Wang Y., Davis G. (2013). The neuropilin 1 cytoplasmic domain is required for VEGF-A-dependent arteriogenesis. Dev. Cell.

[70] Moraes F., Paye J., Mac Gabhann F., Zhuang Z.W., Zhang J., Lanahan A.A., Simons M. (2013). Endothelial cell-dependent regulation of arteriogenesis. Circ. Res..

[71] Moran P., Guo Y., Yuan R., Barnekow N., Palmer J., Beck A., Ren B. (2019). Translating Ribosome Affinity Purification (TRAP) for RNA Isolation from Endothelial Cells In vivo. J. Vis. Exp..

[72] Moran P., Opansky C., Weihrauch D., Yuan R., Jones D.W., Ramchandran R., Ren B. (2017). Abstract 14944: Transcriptional Reprogramming of Endothelial Cells for Arteriolar Differentiation by Small Chemical Molecule via Protein Kinase D1 Signaling Pathway. Circulation.

[73] Wang H.U., Chen Z.F., Anderson D.J. (1998). Molecular distinction and angiogenic interaction between embryonic arteries and veins revealed by ephrin-B2 and its receptor Eph-B4. Cell.

[74] Weinstein B.M., Stemple D.L., Driever W., Fishman M.C. (1995). Gridlock, a localized heritable vascular patterning defect in the zebrafish. Nat. Med..

[75] Zhong T.P., Rosenberg M., Mohideen M.A., Weinstein B., Fishman M.C. (2000). gridlock, an HLH gene required for assembly of the aorta in zebrafish. Science.

[76] Zhong T.P., Childs S., Leu J.P., Fishman M.C. (2001). Gridlock signalling pathway fashions the first embryonic artery. Nature.

[77] Lawson N.D., Scheer N., Pham V.N., Kim C.H., Chitnis A.B., Campos-Ortega J.A., Weinstein B.M. (2001). Notch signaling is required for arterial-venous differentiation during embryonic vascular development. Development.

[78] Weinstein B.M., Lawson N.D. (2002). Arteries, veins, Notch, and VEGF. Cold Spring Harb. Symp. Quant. Biol..

[79] Mukouyama Y.S., Shin D., Britsch S., Taniguchi M., Anderson D.J. (2002). Sensory nerves determine the pattern of arterial differentiation and blood vessel branching in the skin. Cell.

[80] Visconti R.P., Richardson C.D., Sato T.N. (2002). Orchestration of angiogenesis and arteriovenous contribution by angiopoietins and vascular endothelial growth factor (VEGF). Proc. Natl. Acad. Sci. USA.

[81] Hong C.C., Peterson Q.P., Hong J.Y., Peterson R.T. (2006). Artery/vein specification is governed by opposing phosphatidylinositol-3 kinase and MAP kinase/ERK signaling. Curr. Biol..

[82] Peterson R.T., Shaw S.Y., Peterson T.A., Milan D.J., Zhong T.P., Schreiber S.L., MacRae C.A., Fishman M.C. (2004). Chemical suppression of a genetic mutation in a zebrafish model of aortic coarctation. Nat. Biotechnol..

[83] Kazerounian S., Duquette M., Reyes M.A., Lawler J.T., Song K., Perruzzi C., Primo L., Khosravi-Far R., Bussolino F., Rabinovitz I. (2011). Priming of the vascular endothelial growth factor signaling pathway by thrombospondin-1, CD36, and spleen tyrosine kinase. Blood.

[84] Dewey J.F. (1981). Regional tectonics. Science.

[85] Deindl E., Hoefer I.E., Fernandez B., Barancik M., Heil M., Strniskova M., Schaper W. (2003). Involvement of the fibroblast growth factor system in adaptive and chemokine-induced arteriogenesis. Circ. Res..

[86] Aragones J., Fraisl P., Baes M., Carmeliet P. (2009). Oxygen sensors at the crossroad of metabolism. Cell Metab..

[87] Helisch A., Schaper W. (2003). Arteriogenesis: The development and growth of collateral arteries. Microcirculation.

[88] Dong L., Yuan Y., Aguilera-Barrantes I., Chen Y., Sturich A., Yuan R., Wu S., Silverstein R., Ren B. (2015). Abstract 482: Signaling Lipid Lysophosphatidic Acid Is a Critical Link to Diet-induced Obesity, Cellular Bioenergetics and Breast Cancer Angiogenesis. Arterioscler. Thromb. Vasc. Biol..

[89] Yu J.L., Rak J.W. (2003). Host microenvironment in breast cancer development: Inflammatory and immune cells in tumour angiogenesis and arteriogenesis. Breast Cancer Res..

[90] Skuli N., Majmundar A.J., Krock B.L., Mesquita R.C., Mathew L.K., Quinn Z.L., Runge A., Liu L., Kim M.N., Liang J. (2012). Endothelial HIF-2alpha regulates murine pathological angiogenesis and revascularization processes. J. Clin. Invest..

[91] Skuli N., Liu L., Runge A., Wang T., Yuan L., Patel S., Iruela-Arispe L., Simon M.C., Keith B. (2009). Endothelial deletion of hypoxia-inducible factor-2alpha (HIF-2alpha) alters vascular function and tumor angiogenesis. Blood.

[92] Eberhard A., Kahlert S., Goede V., Hemmerlein B., Plate K.H., Augustin H.G. (2000). Heterogeneity of angiogenesis and blood vessel maturation in human tumors: Implications for antiangiogenic tumor therapies. Cancer Res..

[93] Buschmann I., Heil M., Jost M., Schaper W. (2003). Influence of inflammatory cytokines on arteriogenesis. Microcirculation.

[94] Rissanen T.T., Korpisalo P., Markkanen J.E., Liimatainen T., Orden M.R., Kholova I., de Goede A., Heikura T., Grohn O.H., Yla-Herttuala S. (2005). Blood flow remodels growing vasculature during vascular endothelial growth factor gene therapy and determines between capillary arterialization and sprouting angiogenesis. Circulation.

[95] Schechter J., Goldsmith P., Wilson C., Weiner R. (1988). Morphological evidence for the presence of arteries in human prolactinomas. J. Clin. Endocrinol. Metab..

[96] Tinsley J.H., Teasdale N.R., Yuan S.Y. (2004). Involvement of PKCdelta and PKD in pulmonary microvascular endothelial cell hyperpermeability. Am. J. Physiol. Cell Physiol..

[97] Shin D., Garcia-Cardena G., Hayashi S., Gerety S., Asahara T., Stavrakis G., Isner J., Folkman J., Gimbrone M.A., Anderson D.J. (2001). Expression of ephrinB2 identifies a stable genetic difference between arterial and venous vascular smooth muscle as well as endothelial cells, and marks subsets of microvessels at sites of adult neovascularization. Dev. Biol..

[98] Elias D., Lasser P., Ducreux M., Duvillard P., Ouellet J.F., Dromain C., Schlumberger M., Pocard M., Boige V., Miquel C. (2003). Liver resection (and associated extrahepatic resections) for metastatic well-differentiated endocrine tumors: A 15-year single center prospective study. Surgery.

[99] Dromain C., de Baere T., Baudin E., Galline J., Ducreux M., Boige V., Duvillard P., Laplanche A., Caillet H., Lasser P. (2003). MR imaging of hepatic metastases caused by neuroendocrine tumors: Comparing four techniques. Am. J. Roentgenol..

[100] Calderone A. (2018). The Biological Role of Nestin((+))-Cells in Physiological and Pathological Cardiovascular Remodeling. Front. Cell Dev. Biol..

[101] Yazdani S., Kasajima A., Tamaki K., Nakamura Y., Fujishima F., Ohtsuka H., Motoi F., Unno M., Watanabe M., Sato Y. (2014). Angiogenesis and vascular maturation in neuroendocrine tumors. Hum. Pathol..

[102] Hall S.M., Hislop A.A., Pierce C.M., Haworth S.G. (2000). Prenatal origins of human intrapulmonary arteries: Formation and smooth muscle maturation. Am. J. Respir. Cell Mol. Biol..

[103] Helmlinger G., Netti P.A., Lichtenbeld H.C., Melder R.J., Jain R.K. (1997). Solid stress inhibits the growth of multicellular tumor spheroids. Nat. Biotechnol..

[104] Faivre S., Niccoli P., Castellano D., Valle J.W., Hammel P., Raoul J.L., Vinik A., Van Cutsem E., Bang Y.J., Lee S.H. (2017). Sunitinib in pancreatic neuroendocrine tumors: Updated progression-free survival and final overall survival from a phase III randomized study. Ann. Oncol..

[105] Gu J.W., Rizzo P., Pannuti A., Golde T., Osborne B., Miele L. (2012). Notch signals in the endothelium and cancer “stem-like” cells: Opportunities for cancer therapy. Vasc. Cell.

[106] Rafii S., Butler J.M., Ding B.S. (2016). Angiocrine functions of organ-specific endothelial cells. Nature.

[107] Awgulewitsch C.P., Trinh L.T., Hatzopoulos A.K. (2017). The Vascular Wall: A Plastic Hub of Activity in Cardiovascular Homeostasis and Disease. Curr. Cardiol. Rep..

[108] Kohlenberg J.D., Chen Y., Best B., Storz P., Peterson R.T., Silverstein R., Ren B. (2013). Abstract LB-338: A novel LPA-PKD1-FoxO1 pathway in endothelial cells provides an angiogenic switch via down-regulation of CD36 transcription and induction of arteriogenic responses. Cancer Res..

[109] Opansky C., Best B., Yuan R., Cao Q., Ren B. (2016). Protein Kinase D1 Signaling is the Key to Arterial Differentiation of Vascular Endothelial Cells. Circulation.

[110] Yao J.C., Eisner M.P., Leary C., Dagohoy C., Phan A., Rashid A., Hassan M., Evans D.B. (2007). Population-based study of islet cell carcinoma. Ann. Surg. Oncol..

[111] Metz D.C., Jensen R.T. (2008). Gastrointestinal neuroendocrine tumors: Pancreatic endocrine tumors. Gastroenterology.

[112] Lawrence B., Gustafsson B.I., Chan A., Svejda B., Kidd M., Modlin I.M. (2011). The epidemiology of gastroenteropancreatic neuroendocrine tumors. Endocrinol. Metab. Clin. N. Am..

[113] Cueto A., Burigana F., Nicolini A., Lugnani F. (2014). Neuroendocrine tumors of the lung: Hystological classification, diagnosis, traditional and new therapeutic approaches. Curr. Med. Chem..

[114] Liakakos T., Roukos D.H. (2011). Everolimus and sunitinib: From mouse models to treatment of pancreatic neuroendocrine tumors. Future Oncol..

[115] Hori T., Takaori K., Uemoto S. (2014). Pancreatic neuroendocrine tumor accompanied with multiple liver metastases. World J. Hepatol..

[116] Rindi G., Klersy C., Albarello L., Baudin E., Bianchi A., Buchler M.W., Caplin M., Couvelard A., Cros J., de Herder W.W. (2018). Competitive Testing of the WHO 2010 versus the WHO 2017 Grading of Pancreatic Neuroendocrine Neoplasms: Data from a Large International Cohort Study. Neuroendocrinology.

[117] Proye C. (2001). Natural history of liver metastasis of gastroenteropancreatic neuroendocrine tumors: Place for chemoembolization. World J. Surg..

[118] Fendrich V., Waldmann J., Bartsch D.K., Langer P. (2009). Surgical management of pancreatic endocrine tumors. Nat. Rev. Clin. Oncol..

[119] Nguyen S.Q., Angel L.P., Divino C.M., Schluender S., Warner R.R. (2007). Surgery in malignant pancreatic neuroendocrine tumors. J. Surg. Oncol..

[120] Villaume K., Blanc M., Gouysse G., Walter T., Couderc C., Nejjari M., Vercherat C., Cordier-Bussat M., Roche C., Scoazec J.Y. (2010). VEGF secretion by neuroendocrine tumor cells is inhibited by octreotide and by inhibitors of the PI3K/AKT/mTOR pathway. Neuroendocrinology.

[121] Zhang J., Cao R., Zhang Y., Jia T., Cao Y., Wahlberg E. (2009). Differential roles of PDGFR-alpha and PDGFR-beta in angiogenesis and vessel stability. FASEB J..

[122] Raymond E., Dahan L., Raoul J.L., Bang Y.J., Borbath I., Lombard-Bohas C., Valle J., Metrakos P., Smith D., Vinik A. (2011). Sunitinib malate for the treatment of pancreatic neuroendocrine tumors. N. Engl. J. Med..

[123] Valle J.W., Borbath I., Rosbrook B., Fernandez K., Raymond E. (2019). Sunitinib in patients with pancreatic neuroendocrine tumors: Update of safety data. Future Oncol..

[124] Capozzi M., VON Arx C., DE Divitiis C., Ottaiano A., Tatangelo F., Romano G.M., Tafuto S. (2016). Antiangiogenic Therapy in Pancreatic Neuroendocrine Tumors. Anticancer Res..

[125] Berruti A., Fazio N., Ferrero A., Brizzi M.P., Volante M., Nobili E., Tozzi L., Bodei L., Torta M., D’Avolio A. (2014). Bevacizumab plus octreotide and metronomic capecitabine in patients with metastatic well-to-moderately differentiated neuroendocrine tumors: The XELBEVOCT study. BMC Cancer.

[126] Chan J.A., Stuart K., Earle C.C., Clark J.W., Bhargava P., Miksad R., Blaszkowsky L., Enzinger P.C., Meyerhardt J.A., Zheng H. (2012). Prospective study of bevacizumab plus temozolomide in patients with advanced neuroendocrine tumors. J. Clin. Oncol..

[127] Helfrich I., Scheffrahn I., Bartling S., Weis J., von Felbert V., Middleton M., Kato M., Ergun S., Augustin H.G., Schadendorf D. (2010). Resistance to antiangiogenic therapy is directed by vascular phenotype, vessel stabilization, and maturation in malignant melanoma. J. Exp. Med..

[128] Erber R., Thurnher A., Katsen A.D., Groth G., Kerger H., Hammes H.P., Menger M.D., Ullrich A., Vajkoczy P. (2004). Combined inhibition of VEGF and PDGF signaling enforces tumor vessel regression by interfering with pericyte-mediated endothelial cell survival mechanisms. FASEB J..

[129] Sitohy B., Nagy J.A., Dvorak H.F. (2012). Anti-VEGF/VEGFR therapy for cancer: Reassessing the target. Cancer Res..

[130] Uri I., Grozinsky-Glasberg S. (2018). Current treatment strategies for patients with advanced gastroenteropancreatic neuroendocrine tumors (GEP-NETs). Clin. Diabetes Endocrinol..

[131] Raymond E., Kulke M.H., Qin S., Yu X., Schenker M., Cubillo A., Lou W., Tomasek J., Thiis-Evensen E., Xu J.M. (2018). Efficacy and Safety of Sunitinib in Patients with Well-Differentiated Pancreatic Neuroendocrine Tumours. Neuroendocrinology.

[132] Rinzivillo M., Fazio N., Pusceddu S., Spallanzani A., Ibrahim T., Campana D., Marconcini R., Partelli S., Badalamenti G., Brizzi M.P. (2018). Sunitinib in patients with pre-treated pancreatic neuroendocrine tumors: A real-world study. Pancreatology.

[133] Yao J.C., Shah M.H., Ito T., Bohas C.L., Wolin E.M., Van Cutsem E., Hobday T.J., Okusaka T., Capdevila J., de Vries E.G. (2011). Everolimus for advanced pancreatic neuroendocrine tumors. N. Engl. J. Med..

[134] Baldelli R., Barnabei A., Rizza L., Isidori A.M., Rota F., Di Giacinto P., Paoloni A., Torino F., Corsello S.M., Lenzi A. (2014). Somatostatin analogs therapy in gastroenteropancreatic neuroendocrine tumors: Current aspects and new perspectives. Front. Endocrinol..

[135] Caplin M.E., Pavel M., Cwikla J.B., Phan A.T., Raderer M., Sedlackova E., Cadiot G., Wolin E.M., Capdevila J., Wall L. (2014). Lanreotide in metastatic enteropancreatic neuroendocrine tumors. N. Engl. J. Med..

[136] Woltering E.A., Barrie R., O’Dorisio T.M., Arce D., Ure T., Cramer A., Holmes D., Robertson J., Fassler J. (1991). Somatostatin analogues inhibit angiogenesis in the chick chorioallantoic membrane. J. Surg. Res..

[137] Grozinsky-Glasberg S., Shimon I., Korbonits M., Grossman A.B. (2008). Somatostatin analogues in the control of neuroendocrine tumours: Efficacy and mechanisms. Endocr. Relat. Cancer.

[138] Dasgupta P. (2004). Somatostatin analogues: Multiple roles in cellular proliferation, neoplasia, and angiogenesis. Pharmacol. Ther..

[139] Albini A., Florio T., Giunciuglio D., Masiello L., Carlone S., Corsaro A., Thellung S., Cai T., Noonan D.M., Schettini G. (1999). Somatostatin controls Kaposi’s sarcoma tumor growth through inhibition of angiogenesis. FASEB J..

[140] Mentlein R., Eichler O., Forstreuter F., Held-Feindt J. (2001). Somatostatin inhibits the production of vascular endothelial growth factor in human glioma cells. Int. J. Cancer.

[141] Boehm T., Folkman J., Browder T., O’Reilly M.S. (1997). Antiangiogenic therapy of experimental cancer does not induce acquired drug resistance. Nature.

[142] Mauceri H.J., Hanna N.N., Beckett M.A., Gorski D.H., Staba M.J., Stellato K.A., Bigelow K., Heimann R., Gately S., Dhanabal M. (1998). Combined effects of angiostatin and ionizing radiation in antitumour therapy. Nature.

[143] Friedlander M., Brooks P.C., Shaffer R.W., Kincaid C.M., Varner J.A., Cheresh D.A. (1995). Definition of two angiogenic pathways by distinct alpha v integrins. Science.

[144] Ferrara N., Chen H., Davis-Smyth T., Gerber H.P., Nguyen T.N., Peers D., Chisholm V., Hillan K.J., Schwall R.H. (1998). Vascular endothelial growth factor is essential for corpus luteum angiogenesis. Nat. Med..

[145] Han Z.B., Ren H., Zhao H., Chi Y., Chen K., Zhou B., Liu Y.J., Zhang L., Xu B., Liu B. (2008). Hypoxia-inducible factor (HIF)-1 alpha directly enhances the transcriptional activity of stem cell factor (SCF) in response to hypoxia and epidermal growth factor (EGF). Carcinogenesis.

[146] Cooke V.G., LeBleu V.S., Keskin D., Khan Z., O’Connell J.T., Teng Y., Duncan M.B., Xie L., Maeda G., Vong S. (2012). Pericyte depletion results in hypoxia-associated epithelial-to-mesenchymal transition and metastasis mediated by met signaling pathway. Cancer Cell.

[147] Paez-Ribes M., Allen E., Hudock J., Takeda T., Okuyama H., Vinals F., Inoue M., Bergers G., Hanahan D., Casanovas O. (2009). Antiangiogenic therapy elicits malignant progression of tumors to increased local invasion and distant metastasis. Cancer Cell.

[148] Manegold C., Dingemans A.C., Gray J.E., Nakagawa K., Nicolson M., Peters S., Reck M., Wu Y.L., Brustugun O.T., Crino L. (2017). The Potential of Combined Immunotherapy and Antiangiogenesis for the Synergistic Treatment of Advanced NSCLC. J. Thorac. Oncol..

[149] Mack J.J., Iruela-Arispe M.L. (2018). NOTCH regulation of the endothelial cell phenotype. Curr. Opin. Hematol..

[150] Noguera-Troise I., Daly C., Papadopoulos N.J., Coetzee S., Boland P., Gale N.W., Lin H.C., Yancopoulos G.D., Thurston G. (2006). Blockade of Dll4 inhibits tumour growth by promoting non-productive angiogenesis. Nature.

[151] Ridgway J., Zhang G., Wu Y., Stawicki S., Liang W.C., Chanthery Y., Kowalski J., Watts R.J., Callahan C., Kasman I. (2006). Inhibition of Dll4 signalling inhibits tumour growth by deregulating angiogenesis. Nature.

[152] Scehnet J.S., Jiang W., Kumar S.R., Krasnoperov V., Trindade A., Benedito R., Djokovic D., Borges C., Ley E.J., Duarte A. (2007). Inhibition of Dll4-mediated signaling induces proliferation of immature vessels and results in poor tissue perfusion. Blood.

[153] Low S., Barnes J.L., Zammit P.S., Beauchamp J.R. (2018). Delta-Like 4 Activates Notch 3 to Regulate Self-Renewal in Skeletal Muscle Stem Cells. Stem Cells.

[154] Takahashi Y., Akishima-Fukasawa Y., Kobayashi N., Sano T., Kosuge T., Nimura Y., Kanai Y., Hiraoka N. (2007). Prognostic value of tumor architecture, tumor-associated vascular characteristics, and expression of angiogenic molecules in pancreatic endocrine tumors. Clin. Cancer Res. Off. J. Am. Assoc. Cancer Res..

[155] Tan G., Cioc A.M., Perez-Montiel D., Ellison E.C., Frankel W.L. (2004). Microvascular density does not correlate with histopathology and outcome in neuroendocrine tumors of the pancreas. Appl. Immunohistochem. Mol. Morphol..

[156] Laitakari J., Nayha V., Stenback F. (2004). Size, shape, structure, and direction of angiogenesis in laryngeal tumour development. J. Clin. Pathol..

[157] Nayha V.V., Stenback F.G. (2007). Increased angiogenesis is associated with poor prognosis of squamous cell carcinoma of the vulva. Acta Obstet. Gynecol. Scand..

[158] Crabtree J.S., Singleton C.S., Miele L. (2016). Notch Signaling in Neuroendocrine Tumors. Front. Oncol..

[159] Radtke F., Raj K. (2003). The role of Notch in tumorigenesis: Oncogene or tumour suppressor?. Nat. Rev. Cancer.

[160] Kunnimalaiyaan M., Chen H. (2007). Tumor suppressor role of Notch-1 signaling in neuroendocrine tumors. Oncologist.

[161] Kunnimalaiyaan M., Yan S., Wong F., Zhang Y.W., Chen H. (2005). Hairy Enhancer of Split-1 (HES-1), a Notch1 effector, inhibits the growth of carcinoid tumor cells. Surgery.

[162] Kunnimalaiyaan M., Traeger K., Chen H. (2005). Conservation of the Notch1 signaling pathway in gastrointestinal carcinoid cells. Am. J. Physiol. Gastrointest. Liver Physiol..

[163] Kunnimalaiyaan M., Vaccaro A.M., Ndiaye M.A., Chen H. (2006). Overexpression of the NOTCH1 intracellular domain inhibits cell proliferation and alters the neuroendocrine phenotype of medullary thyroid cancer cells. J. Biol. Chem..

[164] Nakakura E.K., Sriuranpong V.R., Kunnimalaiyaan M., Hsiao E.C., Schuebel K.E., Borges M.W., Jin N., Collins B.J., Nelkin B.D., Chen H. (2005). Regulation of neuroendocrine differentiation in gastrointestinal carcinoid tumor cells by notch signaling. J. Clin. Endocrinol. Metab..

[165] Krampitz G.W., George B.M., Willingham S.B., Volkmer J.P., Weiskopf K., Jahchan N., Newman A.M., Sahoo D., Zemek A.J., Yanovsky R.L. (2016). Identification of tumorigenic cells and therapeutic targets in pancreatic neuroendocrine tumors. Proc. Natl. Acad. Sci. USA.

[166] Wang H., Chen Y., Fernandez-Del Castillo C., Yilmaz O., Deshpande V. (2013). Heterogeneity in signaling pathways of gastroenteropancreatic neuroendocrine tumors: A critical look at notch signaling pathway. Mod. Pathol..

[167] Krausch M., Kroepil F., Lehwald N., Lachenmayer A., Schott M., Anlauf M., Cupisti K., Knoefel W.T., Raffel A. (2013). Notch 1 tumor expression is lacking in highly proliferative pancreatic neuroendocrine tumors. Endocrine.

[168] Eliasz S., Liang S., Chen Y., De Marco M.A., Machek O., Skucha S., Miele L., Bocchetta M. (2010). Notch-1 stimulates survival of lung adenocarcinoma cells during hypoxia by activating the IGF-1R pathway. Oncogene.

[169] Sriuranpong V., Borges M.W., Strock C.L., Nakakura E.K., Watkins D.N., Blaumueller C.M., Nelkin B.D., Ball D.W. (2002). Notch signaling induces rapid degradation of achaete-scute homolog 1. Mol. Cell Biol..

[170] Jaskula-Sztul R., Eide J., Tesfazghi S., Dammalapati A., Harrison A.D., Yu X.M., Scheinebeck C., Winston-McPherson G., Kupcho K.R., Robers M.B. (2015). Tumor-suppressor role of Notch3 in medullary thyroid carcinoma revealed by genetic and pharmacological induction. Mol. Cancer Ther..

[171] Somnay Y.R., Yu X.M., Lloyd R.V., Leverson G., Aburjania Z., Jang S., Jaskula-Sztul R., Chen H. (2017). Notch3 expression correlates with thyroid cancer differentiation, induces apoptosis, and predicts disease prognosis. Cancer.

[172] Lou I., Odorico S., Yu X.M., Harrison A., Jaskula-Sztul R., Chen H. (2018). Notch3 as a novel therapeutic target in metastatic medullary thyroid cancer. Surgery.

[173] Zhou M., Jin W.Y., Fan Z.W., Han R.C. (2013). Analysis of the expression of the Notch3 receptor protein in adult lung cancer. Oncol. Lett..

[174] Ito T., Udaka N., Yazawa T., Okudela K., Hayashi H., Sudo T., Guillemot F., Kageyama R., Kitamura H. (2000). Basic helix-loop-helix transcription factors regulate the neuroendocrine differentiation of fetal mouse pulmonary epithelium. Development.

[175] Marcucci F., Caserta C.A., Romeo E., Rumio C. (2019). Antibody-Drug Conjugates (ADC) Against Cancer Stem-Like Cells (CSC)-Is There Still Room for Optimism?. Front. Oncol..

[176] Lashari B.H., Vallatharasu Y., Kolandra L., Hamid M., Uprety D. (2018). Rovalpituzumab Tesirine: A Novel DLL3-Targeting Antibody-Drug Conjugate. Drugs R D.

[177] Saunders L.R., Bankovich A.J., Anderson W.C., Aujay M.A., Bheddah S., Black K., Desai R., Escarpe P.A., Hampl J., Laysang A. (2015). A DLL3-targeted antibody-drug conjugate eradicates high-grade pulmonary neuroendocrine tumor-initiating cells in vivo. Sci. Transl. Med..

[178] Kuhnert F., Kirshner J.R., Thurston G. (2011). Dll4-Notch signaling as a therapeutic target in tumor angiogenesis. Vasc. Cell.

[179] Driessens G., Beck B., Caauwe A., Simons B.D., Blanpain C. (2012). Defining the mode of tumour growth by clonal analysis. Nature.

[180] Schepers A.G., Snippert H.J., Stange D.E., van den Born M., van Es J.H., van de Wetering M., Clevers H. (2012). Lineage tracing reveals Lgr5+ stem cell activity in mouse intestinal adenomas. Science.

[181] Kozar S., Morrissey E., Nicholson A.M., van der Heijden M., Zecchini H.I., Kemp R., Tavare S., Vermeulen L., Winton D.J. (2013). Continuous clonal labeling reveals small numbers of functional stem cells in intestinal crypts and adenomas. Cell Stem Cell.

[182] Zomer A., Ellenbroek S.I., Ritsma L., Beerling E., Vrisekoop N., Van Rheenen J. (2013). Intravital imaging of cancer stem cell plasticity in mammary tumors. Stem Cells.

[183] Chen J., Li Y., Yu T.S., McKay R.M., Burns D.K., Kernie S.G., Parada L.F. (2012). A restricted cell population propagates glioblastoma growth after chemotherapy. Nature.

[184] Oshimori N., Oristian D., Fuchs E. (2015). TGF-beta promotes heterogeneity and drug resistance in squamous cell carcinoma. Cell.

[185] Batlle E., Clevers H. (2017). Cancer stem cells revisited. Nat. Med..

[186] Mathis R.A., Sokol E.S., Gupta P.B. (2017). Cancer cells exhibit clonal diversity in phenotypic plasticity. Open Biol..

[187] Gupta P.B., Fillmore C.M., Jiang G., Shapira S.D., Tao K., Kuperwasser C., Lander E.S. (2011). Stochastic state transitions give rise to phenotypic equilibrium in populations of cancer cells. Cell.

[188] Hoeck J.D., Biehs B., Kurtova A.V., Kljavin N.M., de Sousa E.M.F., Alicke B., Koeppen H., Modrusan Z., Piskol R., de Sauvage F.J. (2017). Stem cell plasticity enables hair regeneration following Lgr5(+) cell loss. Nat. Cell Biol..

[189] Lenos K.J., Miedema D.M., Lodestijn S.C., Nijman L.E., van den Bosch T., Romero Ros X., Lourenco F.C., Lecca M.C., van der Heijden M., van Neerven S.M. (2018). Stem cell functionality is microenvironmentally defined during tumour expansion and therapy response in colon cancer. Nat. Cell Biol..

[190] de Sousa e Melo F., Kurtova A.V., Harnoss J.M., Kljavin N., Hoeck J.D., Hung J., Anderson J.E., Storm E.E., Modrusan Z., Koeppen H. (2017). A distinct role for Lgr5(+) stem cells in primary and metastatic colon cancer. Nature.

[191] Drost J., van Boxtel R., Blokzijl F., Mizutani T., Sasaki N., Sasselli V., de Ligt J., Behjati S., Grolleman J.E., van Wezel T. (2017). Use of CRISPR-modified human stem cell organoids to study the origin of mutational signatures in cancer. Science.

[192] Clevers H. (2015). STEM CELLS. What is an adult stem cell?. Science.

[193] Ball C.R., Oppel F., Ehrenberg K.R., Dubash T.D., Dieter S.M., Hoffmann C.M., Abel U., Herbst F., Koch M., Werner J. (2017). Succession of transiently active tumor-initiating cell clones in human pancreatic cancer xenografts. EMBO Mol. Med..

[194] McGranahan N., Swanton C. (2017). Clonal Heterogeneity and Tumor Evolution: Past, Present, and the Future. Cell.

[195] Jagust P., de Luxan-Delgado B., Parejo-Alonso B., Sancho P. (2019). Metabolism-Based Therapeutic Strategies Targeting Cancer Stem Cells. Front. Pharmacol..

[196] Ryall J.G., Dell’Orso S., Derfoul A., Juan A., Zare H., Feng X., Clermont D., Koulnis M., Gutierrez-Cruz G., Fulco M. (2015). The NAD(+)-dependent SIRT1 deacetylase translates a metabolic switch into regulatory epigenetics in skeletal muscle stem cells. Cell Stem Cell.

[197] Buffie C.G., Bucci V., Stein R.R., McKenney P.T., Ling L., Gobourne A., No D., Liu H., Kinnebrew M., Viale A. (2015). Precision microbiome reconstitution restores bile acid mediated resistance to Clostridium difficile. Nature.

[198] Viale A., Pettazzoni P., Lyssiotis C.A., Ying H., Sanchez N., Marchesini M., Carugo A., Green T., Seth S., Giuliani V. (2014). Oncogene ablation-resistant pancreatic cancer cells depend on mitochondrial function. Nature.

[199] Sancho P., Burgos-Ramos E., Tavera A., Bou Kheir T., Jagust P., Schoenhals M., Barneda D., Sellers K., Campos-Olivas R., Grana O. (2015). MYC/PGC-1alpha Balance Determines the Metabolic Phenotype and Plasticity of Pancreatic Cancer Stem Cells. Cell Metab..

[200] Barnekow N., Yuan R., Moran P., Ren B. (2018). Abstract 16740: Foxo1-Activated Cd36 Transcription Switches Arteriolar Differentiation of Endothelial Cells. Circulation.

[201] Ladanyi A., Mukherjee A., Kenny H.A., Johnson A., Mitra A.K., Sundaresan S., Nieman K.M., Pascual G., Benitah S.A., Montag A. (2018). Adipocyte-induced CD36 expression drives ovarian cancer progression and metastasis. Oncogene.

[202] Ye H., Adane B., Khan N., Sullivan T., Minhajuddin M., Gasparetto M., Stevens B., Pei S., Balys M., Ashton J.M. (2016). Leukemic Stem Cells Evade Chemotherapy by Metabolic Adaptation to an Adipose Tissue Niche. Cell Stem Cell.

[203] Fumagalli A., Drost J., Suijkerbuijk S.J., van Boxtel R., de Ligt J., Offerhaus G.J., Begthel H., Beerling E., Tan E.H., Sansom O.J. (2017). Genetic dissection of colorectal cancer progression by orthotopic transplantation of engineered cancer organoids. Proc. Natl. Acad. Sci. USA.

[204] Fujii M., Shimokawa M., Date S., Takano A., Matano M., Nanki K., Ohta Y., Toshimitsu K., Nakazato Y., Kawasaki K. (2016). A Colorectal Tumor Organoid Library Demonstrates Progressive Loss of Niche Factor Requirements during Tumorigenesis. Cell Stem Cell.

[205] Zhang X., Yalcin S., Lee D.F., Yeh T.Y., Lee S.M., Su J., Mungamuri S.K., Rimmele P., Kennedy M., Sellers R. (2011). FOXO1 is an essential regulator of pluripotency in human embryonic stem cells. Nat. Cell Biol..

[206] Tothova Z., Gilliland D.G. (2007). FoxO transcription factors and stem cell homeostasis: Insights from the hematopoietic system. Cell Stem Cell.

[207] Sykes S.M., Lane S.W., Bullinger L., Kalaitzidis D., Yusuf R., Saez B., Ferraro F., Mercier F., Singh H., Brumme K.M. (2011). AKT/FOXO signaling enforces reversible differentiation blockade in myeloid leukemias. Cell.

[208] Paik J.H., Kollipara R., Chu G., Ji H., Xiao Y., Ding Z., Miao L., Tothova Z., Horner J.W., Carrasco D.R. (2007). FoxOs are lineage-restricted redundant tumor suppressors and regulate endothelial cell homeostasis. Cell.

[209] Wilhelm K., Happel K., Eelen G., Schoors S., Oellerich M.F., Lim R., Zimmermann B., Aspalter I.M., Franco C.A., Boettger T. (2016). FOXO1 couples metabolic activity and growth state in the vascular endothelium. Nature.

[210] Carrasco P., Zuazo-Gaztelu I., Casanovas O. (2017). Sprouting strategies and dead ends in anti-angiogenic targeting of NETs. J. Mol. Endocrinol..

[211] Chu X., Gao X., Jansson L., Quach M., Skogseid B., Barbu A. (2013). Multiple microvascular alterations in pancreatic islets and neuroendocrine tumors of a Men1 mouse model. Am. J. Pathol..

[212] Wagenblast E., Soto M., Gutierrez-Angel S., Hartl C.A., Gable A.L., Maceli A.R., Erard N., Williams A.M., Kim S.Y., Dickopf S. (2015). A model of breast cancer heterogeneity reveals vascular mimicry as a driver of metastasis. Nature.

[213] Hendrix M.J., Seftor E.A., Seftor R.E., Chao J.T., Chien D.S., Chu Y.W. (2016). Tumor cell vascular mimicry: Novel targeting opportunity in melanoma. Pharmacol. Ther..

[214] Zhou L., Liu X.D., Sun M., Zhang X., German P., Bai S., Ding Z., Tannir N., Wood C.G., Matin S.F. (2016). Targeting MET and AXL overcomes resistance to sunitinib therapy in renal cell carcinoma. Oncogene.

[215] Capdevila J., Fazio N., López-López C., Teule A., Valle J.W., Tafuto S., Custodio A.B., Reed N., Raderer M., Grande E. (2019). Progression-free survival (PFS) and subgroups analyses of lenvatinib in patients (pts) with G1/G2 advanced pancreatic (panNETs) and gastrointestinal (giNETs) neuroendocrine tumors (NETs): Updated results from the phase II TALENT trial (GETNE 1509). J. Clin. Oncol..

[216] Capozzi M., De Divitiis C., Ottaiano A., von Arx C., Scala S., Tatangelo F., Delrio P., Tafuto S. (2019). Lenvatinib, a molecule with versatile application: From preclinical evidence to future development in anti-cancer treatment. Cancer Manag. Res..

[217] Grande E., Lopez C., Alonso-Gordoa T., Benavent M., Capdevila J., Teule A., Custodio A., Sevilla I., Gajate P., Molina-Cerrillo J. (2019). The SUNEVO (GETNE-1408) trial to evaluate the activity and safety of thecombination of sunitinib with evofosfamide (TH-302) in patients with G1/G2 metastatic pancreatic neuroendocrine tumours (pNETs) naïve forsystemic treatment: A phase II study of the Spanish Task Force Group for Neuroendocrine and Endocrine Tumors (GETNE). J. Clin. Oncol..

[218] Ikezono Y., Koga H., Akiba J., Abe M., Yoshida T., Wada F., Nakamura T., Iwamoto H., Masuda A., Sakaue T. (2017). Pancreatic Neuroendocrine Tumors and EMT Behavior Are Driven by the CSC Marker DCLK1. Mol. Cancer Res..

[219] Rigamonti N., Kadioglu E., Keklikoglou I., Wyser Rmili C., Leow C.C., De Palma M. (2014). Role of angiopoietin-2 in adaptive tumor resistance to VEGF signaling blockade. Cell Rep..

[220] Biel N.M., Siemann D.W. (2016). Targeting the Angiopoietin-2/Tie-2 axis in conjunction with VEGF signal interference. Cancer Lett..

[221] Maione F., Capano S., Regano D., Zentilin L., Giacca M., Casanovas O., Bussolino F., Serini G., Giraudo E. (2012). Semaphorin 3A overcomes cancer hypoxia and metastatic dissemination induced by antiangiogenic treatment in mice. J. Clin. Invest..

[222] Allen E., Walters I.B., Hanahan D. (2011). Brivanib, a dual FGF/VEGF inhibitor, is active both first and second line against mouse pancreatic neuroendocrine tumors developing adaptive/evasive resistance to VEGF inhibition. Clin. Cancer Res. Off. J. Am. Assoc. Cancer Res..

[223] Hilfenhaus G., Gohrig A., Pape U.F., Neumann T., Jann H., Zdunek D., Hess G., Stassen J.M., Wiedenmann B., Detjen K. (2013). Placental growth factor supports neuroendocrine tumor growth and predicts disease prognosis in patients. Endocr. Relat. Cancer.

[224] Fischer C., Jonckx B., Mazzone M., Zacchigna S., Loges S., Pattarini L., Chorianopoulos E., Liesenborghs L., Koch M., De Mol M. (2007). Anti-PlGF inhibits growth of VEGF(R)-inhibitor-resistant tumors without affecting healthy vessels. Cell.

[225] Marconcini R., Faviana P., Campani D., Galli L., Antonuzzo A., Orlandini C., Falcone A., Ricci S. (2016). Enhancer of zest homolog 2 (EZH2) expression in well and moderately differentiated pancreatic neuroendocrine tumor (pNET). Ann. Oncol..

[226] Yang L., Yu X., Yang Y. (2018). Autotaxin upregulated by STAT3 activation contributes to invasion in pancreatic neuroendocrine neoplasms. Endocr. Connect..

[227] Chen Y., Ramakrishnan D.P., Ren B. (2013). Regulation of angiogenesis by phospholipid lysophosphatidic acid. Front. Biosci. (Landmark Ed).

[228] Liu S., Umezu-Goto M., Murph M., Lu Y., Liu W., Zhang F., Yu S., Stephens L.C., Cui X., Murrow G. (2009). Expression of autotaxin and lysophosphatidic acid receptors increases mammary tumorigenesis, invasion, and metastases. Cancer Cell.

[229] Liu Y., An S., Ward R., Yang Y., Guo X.X., Li W., Xu T.R. (2016). G protein-coupled receptors as promising cancer targets. Cancer Lett..

[230] Zecchin A., Kalucka J., Dubois C., Carmeliet P. (2017). How Endothelial Cells Adapt Their Metabolism to Form Vessels in Tumors. Front. Immunol..

